# The judicial response to rent controls in Europe: Protecting property rights against state’s intervention?

**DOI:** 10.1007/s10657-024-09820-0

**Published:** 2024-10-28

**Authors:** Alessio Sardo, Gianluca Cerruti, Arnulfo Daniel Mateos Durán, Allegra Grillo

**Affiliations:** University of Genoa, Genova, Italy

**Keywords:** Rent controls, Property rights, Tenant’s protections, Legal reasoning, Housing, K10, K11, K12

## Abstract

This essay examines, from a legal and economic perspective, the judicial response to rent controls in the EU focusing on three courts that operate at the fundamental rights and constitutional level: the European Court of Human Rights (ECtHR), the German Federal Constitutional Court (BVerfGE), and the Italian Constitutional Court. Based on an analysis of a sample of judicial decisions rendered over time, a convergent trend emerges: these Courts have recognized and effectively protected the landlord’s property rights against rent controls that were disproportionate and could not ensure a reasonable return on investment. This trend is prominent in the jurisprudence of the ECtHR: the Strasbourg Court has contributed to reshaping the distribution of power between tenants and landlords, encouraging the transition of Eastern and Southern European Countries to the common European housing market. In both upholding and striking down rent control measures, judges generally take market value and the comparative reference price as the preferred benchmarks for fair rent price. A consumption-based and financial characterization of housing, coupled with the fundamental right to derive economic benefits as core elements of the right to property, underpin the legal reasoning of the three Courts.

## Introduction

European governments are still relying on rent regulations to ensure contractual stability and affordable housing in tight real estate markets (Jones Day, 2020). While activists and several policy makers view rent controls as a quick way of promoting redistributive goals and means to achieve *price equity* (Been et al., 2019; Gyourko & Linneman, 1989), many economists have often portrayed rent controls as inefficient, as these measures distort the spontaneous market dynamics (Frankena, 1975; Fraser Institute, 1975; Olsen, 1988, 1998; Albon & Stafford, 1990; Ault & Saba, 1990; Ho, 1992; Arnott, 1995; Kutty, 1996; Glaeser, 2002, 2013; Glaeser & Luttmer, 2003; Jenkins, 2009). Most EU governments take an intermediate stance: regulating the housing price seems necessary for either preventing housing shortage, or introducing new focal points and equilibria in dysfunctional housing markets that generate pressure on other sectors: health, labor, and education.

Clearly, rent controls have an impact on the housing market performance and households’ choices. For instance, changes in rent prices can produce shifts in first house buyers’ utility functions (Basu & Emerson, 2003). Deciding whether to become first house buyer is a decision dependent on both the budget constraints, and the comparison between rent price and accessibility of a mortgage (Basu & Emerson, 2000, 2003).

In addition, several economists have explored the distorting implications of rent controls, suggesting that these regulations may exert negative spillover effects on neighbouring housing markets, particularly on the long run (Autor et al., 2014; Sims, 2007). Basu and Emerson (2000) emphasize the redistribution effect of rent controls between new tenants and “incumbents” (*i.e*., tenants who have been renting for an extended period). This phenomenon might induce landlords to proactively raise rental costs to offset the erosion of future rents.

According to many economists, the distorting effects of rent controls may extend to discouraging property maintenance by landlords. Moreover, stringent regulations, given the reduced net returns on investments, could deter the construction of new apartments (Arnott et al., 2003; Sims, 2007). Additionally, rent caps might incentivize property owners to reconfigure apartments to be exempt from regulation, resulting in a detrimental impact on the supply of rental housing (Diamond et al., 2019).

Other scholars highlight how rent regulations can curtail resident mobility, thereby diminishing their incentives to relocate (Diamond et al., 2019; Lind, 2001; Nagy, 1997). In general, the traditional economic theory until a few decades ago posits that rent controls diminish incentives for investments and, in the long run, exacerbate housing shortages. These unintended and undesirable consequences—which diminish the net benefit of rent control—underscore the importance for policymakers to consider a wide range of effects and their interactions when designing rent control policies.

Among others, Mense et al. (2023) and Breidenbach et al. (2022) assessed the impact of second-generation rent control in Germany, finding a reduction in rents and home prices in the regulated sector and a simultaneous increase in home prices in the free market, along with reduced maintenance efforts after the cap announcement. Arnott (1995) advocated for a reassessment of soft rent control systems, particularly second-generation rent controls, which, being highly heterogeneous, should be evaluated on a case-by-case basis. In fact, second-generation regulations are more flexible and, when designed appropriately, can enhance welfare (Arnott, 1995).

At any rate, rent controls impose strong—and, as we shall see, often disproportionate—limitations on the core elements of the right to property: the right to derive economic benefits (*ius fructi*) and the right to use and abuse of the property (*ius abutendi*). Rent controls are unconstitutional when they violate the fundamental right to property, imposing excessive burdens on ownership.

This paper examines the judicial response to rent controls in the EU. Based on an analysis of a sample of judicial decisions rendered over time across Europe, a convergent trend emerges. *Interpretive analysis* suggests that a robust conception of property rights protection combined with a market-based approach underpins the judiciary’s assessment of rent controls. European courts, after all, have protected the fundamental rights of landlords. This trend is prominent in the jurisprudence of the European Court of Human Rights (ECtHR): the Strasbourg Court has contributed to reshaping the distribution of power between tenants and landlords, favoring the latter in the transition of both Southern and Eastern European Countries to the EU common market. Starting from the 1980s, European courts have progressively invalidated state-imposed rent freezes and other strict rent control regulations when these measures did not ensure to landlords a reasonable return on investment, or the right to regain possession of their property. In doing so, the judiciary has consistently protected the right to property at a constitutional level and clearly rejected, firstly, the concept of a redistributive or social utility function inherent in the right to property and, secondly, the claim that housing is a positive right.^1^

One point should be emphasized from the outset: the fact that rent control issues have reached in the last decades several EU constitutional courts and the ECtHR, too, means that the definition of the right to housing—both in terms of content and procedural protection—is an issue that affects fundamental rights and freedoms. In other words, the right to housing has, over time, acquired a constitutional dimension that has to do with the fundamentals of life. As our contextual analysis will show, this is by no means coincidental.

The essay is structured as follows. Section 2 contains details on the methodology employed and the justification of the case studies selection; Sect. 3 defines rent controls as complex legal frameworks. Getting to the core of the paper, Sec. 4 explores representative decisions of the ECtHR. Section 5 deals with the jurisprudence of the Italian Constitutional Court, considering its relationship with the trends of the Italian Cassation Court. Section 6 explores the jurisprudence of the German Federal Constitutional Court (BVerfGE) on rent controls, analyzing in greater detail the recent Berlin’s *Mietendekel* case, which prompted a return to discussion on the topic in the EU. Section 7 discusses the right to property vis-à-vis State interventions on rent price. Section 8 proposes a contextual analysis that shows how tenants have come to face repeated housing affordability crises, a phenomenon that explains the expansion of the jurisprudence of the three courts on rent controls. Section 9 contains concluding remarks. The essay includes data visualizations and statistics that support the claims raised by the authors and an Appendix with further figures, data, and calculations.

## Methodology

This research summarizes the results of an inquiry that considered over 200 cases on rent controls taken from the ECtHR, the Italian Constitutional Court, the German Federal Constitutional Court (BVerfGE), and the Italian Cassation Court. The sample of cases was obtained through a semantic analysis of case law on the major databases using keywords that denote rent controls and cognate concepts. Crossed-check controls have been performed by looking at the pertinent academic literature (Fraser Arnott, 1995; Ault & Saba, 1990; Fallis, 1988; Hubert, 1993; Institute, 1975; Turner & Malpezzi, 2003), repositories, and grey literature (*e.g*., European Parliament, 2020; Feantsa, 2021; OECD, 2019, 2021). These cases represent the entire population of cases on rent controls decided by the Courts analyzed in this pilot study.

As far as the ECtHR is concerned, we found 450 cases on housing rights in the HUDOC database: 50 of them dealt with rent regulations. Similarly, we isolated 40 cases of the Italian Constitutional Court by performing a semantic analysis by keywords in the pertinent databases (Corte Costituzionale’s official website, OneLegale, DeJure, Smart24Lex). Finally, we reviewed the case law cited by Italian legal scholars in the main treatises on rent regulations (See e.g., Bargelli & Bianchi, 2018a, 2018b; Calderai, 2012). It was quite difficult to pinpoint the entire jurisprudence of the Italian Court of Cassation. The Constitutional Court issued 300 judgments in the period 2009–2023 (Nevola & Verrengia, 2023), while the Civil Law Section of the Cassation Court has decided an average of 31,000 cases per year (Ufficio di Statistica della Corte Suprema di Cassazione, 2023). The Statistical Yearbook of the Court of Cassation reports the absolute value of decisions on residential leases, which amounted to 940 decisions taken between 2014 and 2023 (Ufficio di Statistica della Corte Suprema di Cassazione, 2023). Considering the total number of Cassation judgments, we identified the relevant decisions through semantic research in the above-mentioned databases and by reviewing the case law cited in the major law journals.

Finally, a reference to the German Federal Constitutional Court is in order. The German Federal Constitutional Court publishes its most important decisions in anthologies entitled “*Entscheidungen des Bundesverfassungsgericht*s (BVerfGE)”. Within these collections, an alphabetical index of the subject matter of each decision (*Sachregister*) is published every ten volumes. These alphabetical indices report 43 cases on rent controls. We have also consulted the main repositories (*e.g*., the “*Nachschlagewerk der Rechtsprechung des Bundesverfassungsgerichts*”) to search for further cases. We then used the cross-references in each case retrieved to find more precedents.

After obtaining a first list of cases, further exclusion criteria have been set, considering that the focus of this research is on the *legal validity* of rent controls and the *social function of property*, *the extensive or restrictive* interpretations of these measures, and their *impact on tenant’s protections*. Many cases decided by the above-mentioned courts dealt with general legal issues that go beyond the scope of this inquiry, such the retroactivity of law. The representative sample was further reduced to 90 key cases.^2^

Interpretive analysis was then applied to these cases to isolate the main arguments employed by the European judiciary for assessing rent control regulations. The study has focused also on the specific parameters chosen for assessing the cases (*e.g*., equality clause, legitimate aim test, property clause) as well as on the intensity of the measure under evaluation. The research is a *pilot study* that fills *a wide gap in the literature*: although rent controls and their economic impact have been largely explored (see e.g., Colomb & De Souza, 2021; Mense et al., 2023; Thies, 1993; Turner & Malpezzi, 2003), a systematic study on the treatment of these regulations by the judiciary is still missing (see *above,* para. 1).

When dealing with the judicial response to rent controls, one must start with the decisions of the Constitutional Courts, Human Rights Courts, and High Courts, as these institutions are empowered to determine the *constitutionality* of the policies endorsed by national governments in their respective jurisdictions or check whether the government’s action violated fundamental rights. The decisions of these courts are *binding precedents* for the lower courts. When we look at the jurisprudence of the Constitutional and Cassation Courts, we must consider their case selection mechanisms: the Italian Constitutional Court, for example, only rules on the constitutionality issues raised by lower courts within a doubtful case; the ECtHR, instead, decides on Human Rights claims of applicants who have exhausted the “internal” (*i.e*., national) remedies. Unlike the U.S. Supreme Court, all the Courts analyzed in this essay have no discretion in the selection of cases or the composition of the docket. However, it is likely that these Courts will dismiss irrelevant claims either on formal grounds, or for “obvious unfoundedness” (*see e.g*., the so-called “*dichiarazione di manifesta infondatezza*” of the Italian Constitutional Court). Due to data protection regulations (e.g. GDPR, Reg. (UE) 2016/679) and procedural rules, it is impossible to obtain data on the income of the applicants, or private information concerning the parties involved in the case.

The courts selected for this pilot study have both similarities and differences. On the one hand, they all belong to the civil law tradition; on the other hand, the courts differ in procedures, formal outcomes (*e.g.,* different types of inadmissibility), and composition. For example, there are essentially three methods for “activating” the constitutional review of legislation (Bin & Pitruzzella, 2023; Steinberger, 1993): (A) the complaint is filed by public or constitutional institutions, a parliamentary group, or a regional government; (B) when ordinary judges have to decide a particular case, they can raise the issue of the constitutionality of a law; (C) individuals can file a direct constitutional complaint to the Constitutional Court if they consider that their fundamental rights or freedoms have been violated. The Italian Constitutional Court can only be reached by individuals via A (“indirect” or “cross” appeal); the BverfGE can be reached via A, B and C; finally, the ECtHR hears cases based on C.

We have selected the Italian Constitutional Court and the BverfGE also because they belong to countries that display significant differences at the socioeconomic level with an eye to the housing market. For instance, whereas in Italy the homeownership rate is higher that 73%, in Germany is only slightly above 46% (EUROSTAT, 2023). The Italian economic system is radically distinct from the German: the former is mainly a familistic welfare regime, whereas the latter is a corporativist welfare system (Arbaci, 2019). There are further cultural and demographic differences between the two States. The ECtHR was included because it is a human rights court with wide territorial jurisdiction (extending to the territories under the effective control of the High Contracting Parties that have signed the ECHR). Moreover, the judges at the Strasbourg Court differ greatly in terms of age, nationality, gender, and legal culture.^3^ Finally, the introduction of the Court of Cassation has been useful to examine whether there is consistency or tension between the two highest legitimizing bodies within a given country. The finding that common patterns are present (with contained variance), even when the procedural framework and socioeconomic system shifts, strengthens the claim that these are not purely contingent and contextual, or at least dependent on formal legal aspects. Future research could involve a higher number of European constitutional courts and the European Court of Justice.^4^

In Sect. 8, leveraging data from various national and international sources—with specific reference to Italy, Germany, and the EU27—we have presented descriptive evidence of certain trends that have occurred concurrently with the growth of the jurisprudence on rent controls in the EU.

## Rent controls as complex and heterogeneus regulatory frameworks

### Generations of rent controls

Rent controls were first introduced in Europe and worldwide as emergency measures after WWI and WWII (*First generation rent controls*) (see e.g., Arnott, 1995 p. 100; Dinse, 2015; Jenkins, 2009 p. 74; Kholodilin, 2024). These policies aimed at containing the shortage of affordable housing, produced by the lack of housing units after the war destruction and mitigate the drawbacks of the slow reconstruction phase, in a context characterized by scarce recourses and limited central planning. Rent controls reemerged in the 1970s to counter the drawbacks of high inflation rents, the oil and energy crisis, and the economic stagnation that affected Western Countries (Iannello, 2022). Today, most European systems rely on the so-called *second* and *third generation rent controls,* which are complex normative frameworks that protect tenants from excessive rents but, at the same time, must ensure a *reasonable return on investment for landlords* (Jenkins, 2009; TENLAW 2015: Norberg & Juul-Sandberg, 2016, 2018; Korthals Altes, 2016).^5^ Second and third generation rent controls aim at striking a balance between the property rights of the landlord, and the protection of tenants.^6^ In this respect, the rent cap (*Mietendeckel*) enacted by the Senate of Berlin to deal with the increasing rent prices deserves particular attention, as it deviates from the European trend. This regulation was similar to a first-generation rent control, imposing a rent freeze for 5 years. The *Mietendeckel* was declared unconstitutional by the BVerfGE: Sect. 6.3 below offers a detailed analysis of the case.

Housing market regulations vary across Europe, and the specifics can differ from country to country or even within different regions of a country (TENLAW, 2015; Jones Day, 2020; OECD, 2021; Feantsa, 2021). Most systems have a *dual regime*: a non-regulated market is coupled with a rent-controlled market. For instance, both Sweden and Denmark rely on rent controls: however, whereas the Danish system applies a dual regime to the housing stock, in Sweden almost all rental units are subject to the same price regulation (Norberg & Juul-Sandberg, 2016). In Germany, instead, while parties are in principle free to set the initial rent through free bargaining, a rent break (*Mietpreisbremse*) applies in specific areas characterized as “tense” or “tight” housing markets (*angespannter Wohnungsmarkt*). The German “rent break” was first introduced in 2015 and then, amended in 2020, as an emergency measure in force for maximum 5 years if not further extended. In particular, the *Mietpreisbremse* imposes constraints rent increases. The maximum rent is typically set as a percentage slightly above the local reference market rent. If a new residential lease is concluded within an area with a “tense housing situation”, the following conditions apply: (A) the rent must not be higher than the 10% of the *standard comparative rent* or, in any case, it must be parametrized to specific indicators; (B) the rent cannot increase by more than 15% (or, in some areas, 20%) in less than three years and, in any case, the increase cannot be set as to exceed the comparative rent by more than 10% (BGBl. 2015 I Nr. 16: 610–612; BGBl I Nr. 14: 540). In the case of existing rental agreements, the landlord can request an increase in the rent up to the local comparative rent (*ortsübliche Vergleichmiete*), when the rent has remained unchanged for at least 15 months and this rent may not increase by more than 20% in the following three years (Section  588, Paragraph 3 BGB). In case of a “tight housing situation”, the maximum increase percentage can be reduced to 15%.^7^

In Italy, a free bargaining system, and a system of rent stabilization (“*canone concordato*”) coexist. The so-called *Fair Rent Act* (L. 392/1978) and the 1998 *Rent Reform Act* (L. 431/1998) set the general legal framework for private houses rents. The *Fair Rent Act* contains a rent stabilization mechanism: the prohibition of increasing the rent after the first four years of an amount that exceeds the 75% of the cost of living. What is more, landlords are not entitled to ask the tenant to cover more than three months of rent in advance. The framework includes the so-called “special” or “conventional” rent contracts. These contracts have a legal duration which is inferior to the standard “four plus four years” contract and are subject to a *low rent price cap*, established through collective agreement between landlords and tenants’ associations.

In the 1970s and early 1980s, Italy had endorsed a more protective legal framework*,* which was replaced by L. 431/1998 and the L. 164/2014. The old normative framework (*equo canone*) granted rent stabilization through a cap for the rent price, which was not supposed to trespass 3,85% of the rental value; the rental value, in turn, was indexed to a set of context-sensitive parameters (including the dimension of the city, the region, the physical condition of the building, and the dimensions of the dwelling). The 1998 reform introduced a new scheme that was less favorable to the tenants: the contractual freedom of the parties is now unconstrained, and the landlord can increase the rent within the broad limit of 75% of the ISTAT index. What is more, the 1998 reform introduced a new regulation on tax evasion to reduce negative externalities in the housing market (Bargelli & Bianchi, 2018a, 2018b). As a response to the judiciary’s stimuli, the Italian legislator has reduced the intensity of rent regulation over time, moving towards second and third generations rent controls that allowed landlords to obtain a reasonable return on investment (more on this *below*).

These are only few examples of rent controls in the EU. Another example of low intensity rent regulation is Llei 11/2020 (18 September) on rent controls in Cataluña, which was also the object of constitutional review (*See generally* Kholodilin, 2024).^8^

### Breaking down the regulatory framework

To keep housing costs affordable without burdening excessively landlords’ interests, policymakers have improved rent control regulations, transforming them from simple rent caps (first generation rent controls) into more complex normative frameworks (Iannello, 2022; Jenkins, 2009; Schmid, 2018)^9^: second- and third- generation rent controls. The complexity of these regulations is a byproduct of the dual aim pursued by the new regulations: on the one hand, the aim of reducing both the price-setting power of landlords and uncertainty in housing markets for tenants; on the other hand, the aim of protecting the right to a reasonable return on investment of landlords, keeping it as close as possible to the market value. For example, many rent freeze regulations were replaced by indexation systems that, in turn, required adjustment mechanisms and oversight boards.

The elements of a typical or rent control regulatory framework include^10^:

1. *A rent ceiling or a controlled rent price.* Rent price is normally established according to a benchmark range or a specific index, as explained above.
2. *Rent increase limitations.* Most rent controls place limitations on the annual rent increase for properties located in designated “stressed areas.” These areas typically have a tight housing market with high demand and limited supply. Legal systems fix the rent increase limit either through a negotiation of the representative organizations of tenants and landlords, or as a function of a particular index. For instance, the price of a rent might change in function of the Consumer Price Index (CPI), which, in turn, measures changes in the cost of living by tracking the average price of a basket of goods and services.
3. *The domain of application.* Rent controls normally apply only to a portion of the housing stock. As hinted above, two normative frameworks, one of controlled rent and one of free market negotiation, can coexist within a same legal system. In Italy, for instance, there are three standard lease types: type (A) 3 plus 2 years agreed rent contract; type (B) 4 plus 4 yeas free rent contract; type (C) 1–18 months transitional lease/ and 6–36 months students rent. Provided that the choice is up to the parties, the portion of housing stock subjected to rent controls can vary significantly over time.
4. *The timeframe of application.* Rent control measures are often implemented for a limited period, typically for three or five years. This is meant to balance the interests of both tenants and landlords, without introducing excessive burdens on landlords. The limited timeframe is tied to the notion that rent controls are still regarded as *emergency regulations* by most EU legal systems.
5. *An oversight board.* Several regulations endorse a rent oversight board or rent stabilization board. Spain, for instance, has established a Public Rents Registry to monitor and control rental prices in stressed areas. Landlords are required to register rental contracts, and rental price increases are subject to regulatory scrutiny.
6. *A list of exceptions*. Rent controls allow for exceptions. *First*, not all areas are subject to rent control measures, and the laws provide for flexibility in determining which regions or neighborhoods are designated as stressed areas. *Second,* not every apartment within a rent-controlled area is subject to regulation: for instance, luxury apartments can have higher rents.
7. *Enforcement mechanisms and penalties*. Local housing authorities are responsible for enforcing rent control regulations, and landlords who violate these regulations may face fines.

**Fig. 1:**
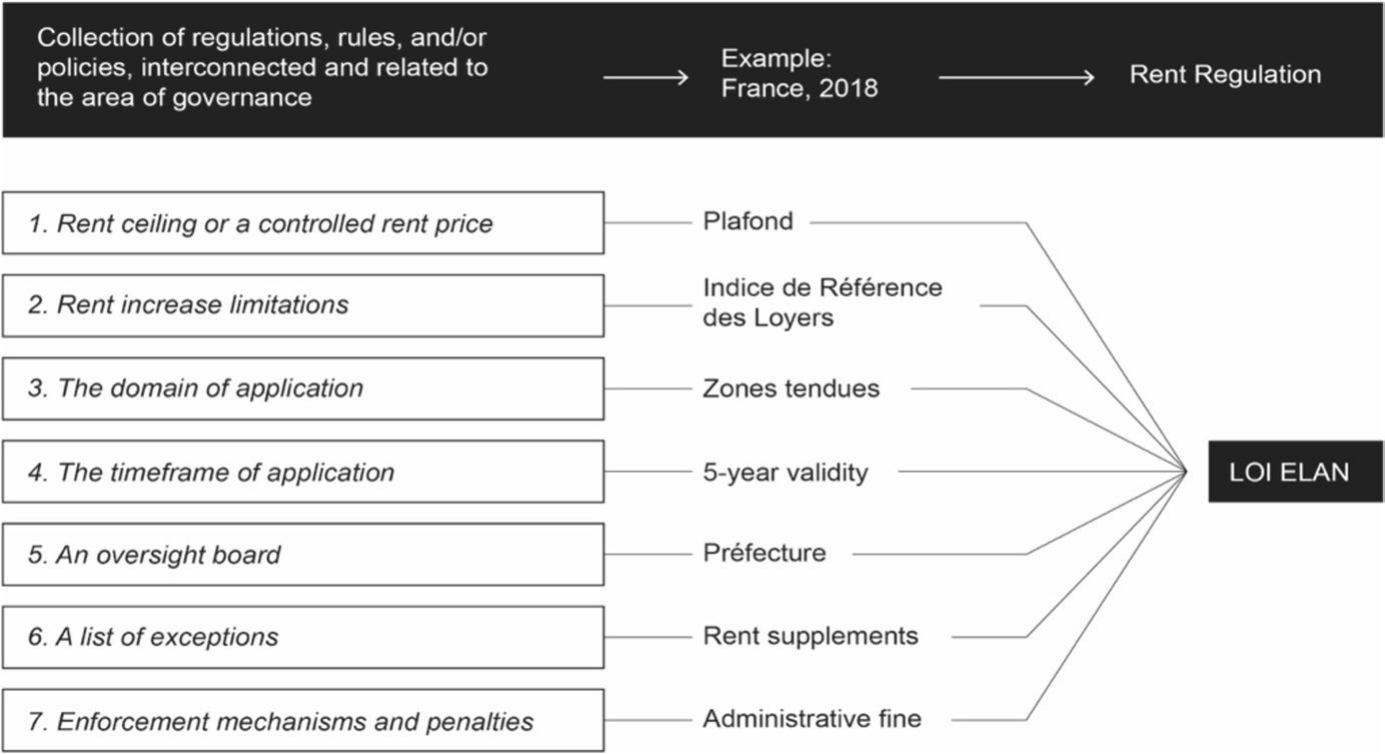
Law Elan system. Compiled by the authors

The following diagram (Fig. 1) offers a typical example of a rent control regulation, introduce in France by the *Loi Elan* (2018):

It is worth noting that rent controls, like all public policies, have both intended and *unintended* effects. A valuable exercise in assessing the impact of rent control is provided by Kholodilin (2024), who examined a substantial body of empirical studies (112 papers) on rent control published in refereed journals over the past 60 years, from 1967 to 2023. Although a significant portion (73% of the studies) pertains to the United States and only about 12,5% of the papers considered in this literature review focus on Germany and less than 1% on Italy—the main countries of our analysis—it is still useful to summarize these findings, which present a mix of positive and negative results.

On the positive side, Kholodilin (2024) highlights the consistent finding that rent control effectively caps rents: 36 out of 41 published papers (and 53 out of 60 papers, including unpublished ones) indicate that rent control achieves its primary objective of rent stabilization. Furthermore, most of the few studies that do not find significant effects employ linear regressions and at least half focus on first-generation rent controls.

On the negative side, Kholodilin (2024) also underscores the unintended consequences of rent control: the majority of studies highlight some negative outcomes, such as increased rent prices for uncontrolled units, decreased mobility (with potential adverse effects on the labor market), and a decline in the quality of housing for rent-controlled dwellings. The impact on homeownership remains unclear.

These unintended consequences—which diminish the net benefit of rent control—underscore the importance for policymakers to consider a wide range of effects and their interactions when designing rent control policies. Constitutional Courts and the ECtHR check whether the trade-offs of the respect the fundamental rights of both landlords and tenants.

## The ECtHR: supporting the transition to the EU common market

### The General Framework

The ECtHR normally decide cases involving rent controls—and, in general, housing regulations—under Article 8 (right to privacy) and Article 1 Par. 1 of the Convention (protection of property and protection against interference with the peaceful enjoyment of possession).^11^ Instead, the Court seems reluctant to adjudicate rent control cases under Article 14 of the ECHR (the equality clause), which is at best considered superfluous when invoked by applicants.^12^ Thus, the Strasbourg judges are very careful not to link rent controls to equality arguments. Moreover, the ECtHR does not derive a positive right to affordable housing from the Convention.^13^ This trend also depends on the political and social context of the cases: many ECtHR decisions evaluate rent control measures that were approved by the socialist regimes of Eastern European or strong redistributive regimes of the Mediterranean region and were still in force during the transition to a free market economy in the housing sector, imposing excessive burdens on ownership.^14^ In all these cases, the ECtHR systematically overturns rent controls for favoring the development of a common European real estate market, particularly when the national judiciary was resisting this socioeconomic change (*see infra,* Sect. 8).

The applicants are mostly landlords challenging the rent ceiling, claiming the impossibility to recover possession (within a reasonable time), and denouncing the length of the eviction proceedings. Pecuniary damages are tied to a loss of profit. Landlords (almost) always win,^15^ obtaining both pecuniary and non-pecuniary damages.^16^ The ECtHR emphasizes the generality and temporality requirement of rent controls,^17^*taking the market value as the benchmark for a fair or reasonable price*,^18^ and exploring counterfactually the difference between the actual rent price under the rent control scheme and the rent that the landlord “would have been likely to obtain for his property on the open market”.^19^ Under the Court’s construction, the landlord’s right to property and protection against interference with the peaceful enjoyment of possession presupposes the right to rent dwellings at their market value, not at a lower price, and this equation strikes a fair balance between the demands of the general, social interest of the State, and the protection of the individual’s fundamental rights enshrined in the Convention.^20^ By the same token, the claim that rent controls are necessary for the “peaceful possession” of the tenants are normally rejected.^21^ The “mismatch between the Government policy plans which envisaged the introduction of market-level rents and the actual lack of progress in that direction” becomes a “factor in assessing the proportionality of the rent control measures which affected the applicants.”^22^

It is worth noticing that the ECtHR generally assumes that rent control has the constitutive effect of producing pecuniary and non-pecuniary damages, taking cues from the equation “pecuniary loss = market value—rent control price”.^23^ The market value is determined through counterfactual reasoning by the ECtHR. With this in mind, let us explore more deeply some striking examples of the ECtHR jurisprudence on rent controls.

### Landmark decisions

In the authoritative precedent *Hutten-Czapska v. Poland* (2006), the ECtHR sided with the landlords. The applicant was the owner of a property that fell under a “special rent regulation” (*szczególny tryb najmu*) introduced in 1974, which was then extended in the late 1990s due to the housing shortage in Poland. The controlled rent imposed by the lease was lower than the cost of maintaining the residential buildings, resulting in a deficit that was covered by the landlords. The Applicant claimed that the Polish authorities had deprived her parents and herself of any opportunity to live in their own home and sought damages for decay of the property and arbitrary change of its use, as well as for psychological distress. The Applicant argued that the Polish authorities had failed to provide adequate compensation or restitution for the property and had denied her requests for eviction orders against the (disabled) tenants.

The argument points out that the rent price was not sufficient to cover the necessary maintenance and taxes.^24^ The ECtHR relies on the standard of “net market rent” to quantify the reasonable return on capital (§ 202; § 242) and finds that Poland violated Article 1 of Protocol No. 1 to the European Convention on Human Rights, which protects the right to property (§§ 223–225). Damages, too, were calculated based on net market rent (rather than maintenance costs). The ECtHR unanimously found that the landlord’s burden was “disproportionate”, and that Poland had not provided the applicant with an effective remedy to resolve her property-related complaints. The overall judgment in this case emphasized the importance of the State’s obligation to protect property rights and provide effective remedies in property dispute cases (§§ 167–168; 296–200).

In the landmark case of *Bittó and Others* (2014), the ECtHR found that the residual rent controls imposed by the Slovak State in some areas did not create a fair balance (§ 116) and placed the entire burden on a small social group: a limited number of landlords. The controlled rent was too low because it corresponded to 26% of the market value. Conversely, the gap between rent and value was too high, and this was considered a serious interference with property rights, a violation of Article 1 of Protocol 1, and a lack of a fair balance (§§ 133–135). The standard of assessment in this case is clearly the ratio between controlled rent/market rent, which was used for estimating the appropriate compensation, too.^25^ As in *Zammit* (§§ 48–52), the Court acknowledges that the price should arise from a free bargaining between landlords and tenants.

Similarly, in *Anthony Aquilina* (2014), the Strasbourg Court held that rent control enforced by the Maltese State was a disproportionate burden on unit owners who were forced into an open-ended landlord-tenant relationship. Because of the combination of low rents and long-term contracts, the possibility of tenants voluntarily moving out was very low, resulting in a state of insecurity that interfered with the property rights of landlords protected by Article 1 Protocol 1. Once again, the market value of rent was used as the standard for assessing the reasonableness of the rent price and the existence of a disproportionate burden on property rights.

The market-oriented approach is also present when the ECtHR upholds a regulatory bundle on rent control, as in *Mellacher & Others* (1989). In that case, the ECtHR ruled that Austria had not violated Article 1 of Protocol No. 1 in the three joint cases challenging the 1981 *Rent Control Act* in Graz. While there was clearly an interference with property rights, the rent reduction determined by the Graz Arbitration Board corresponded to just compensation because it was not disproportionate considering the market value of the housing units. In this case, too, an assessment under Article 14 was deemed superfluous by the ECtHR.

### The denationalization phase

The cases explored in Sect. 4.2 can be readily explained in the context of a socio-economic transition of legal systems with high tenants’ protections and strong limitation of landlords right to a market-based housing economy. The “denationalization” phase of Eastern European Countries is neatly reconstructed in the *obiter dicta* of *Berger Krall & Others v. Slovenia.* Southern and Central European Countries, too, started a phase of privatization of public assets previously mostly subtracted to the free market, including the housing stock. In Italy, for instance, the gradual privatization of public companies and the simultaneous liberalization of economic sectors started in the 1990s under Law 35/1992. This process, propelled by an increasingly urgent need for the recovery of public finances, facilitated the transformation of prominent State-owned enterprises, notably IRI, ENI, INA, and Enel, into joint-stock companies (refer to Decree 333/1992). The ongoing process extends across various sectors, encompassing energy, transportation, banking, telecommunications and, ultimately, housing.

In a parallel vein, Germany underwent a privatization process triggered by the reunification of the country in 1990 under the *Einigungsvertrag*. The explicit objective was to convert inefficient State-owned enterprises into efficient and competitive entities operating within a market economy, as outlined in the *Treuhandgesetz*. In both instances, the management of transitions and the social impacts of privatizations prompted controversies and debates: to be sure, the former regulations introduced by the socialist parties touched upon the very core of the right to ownership.

**Fig. 2:**
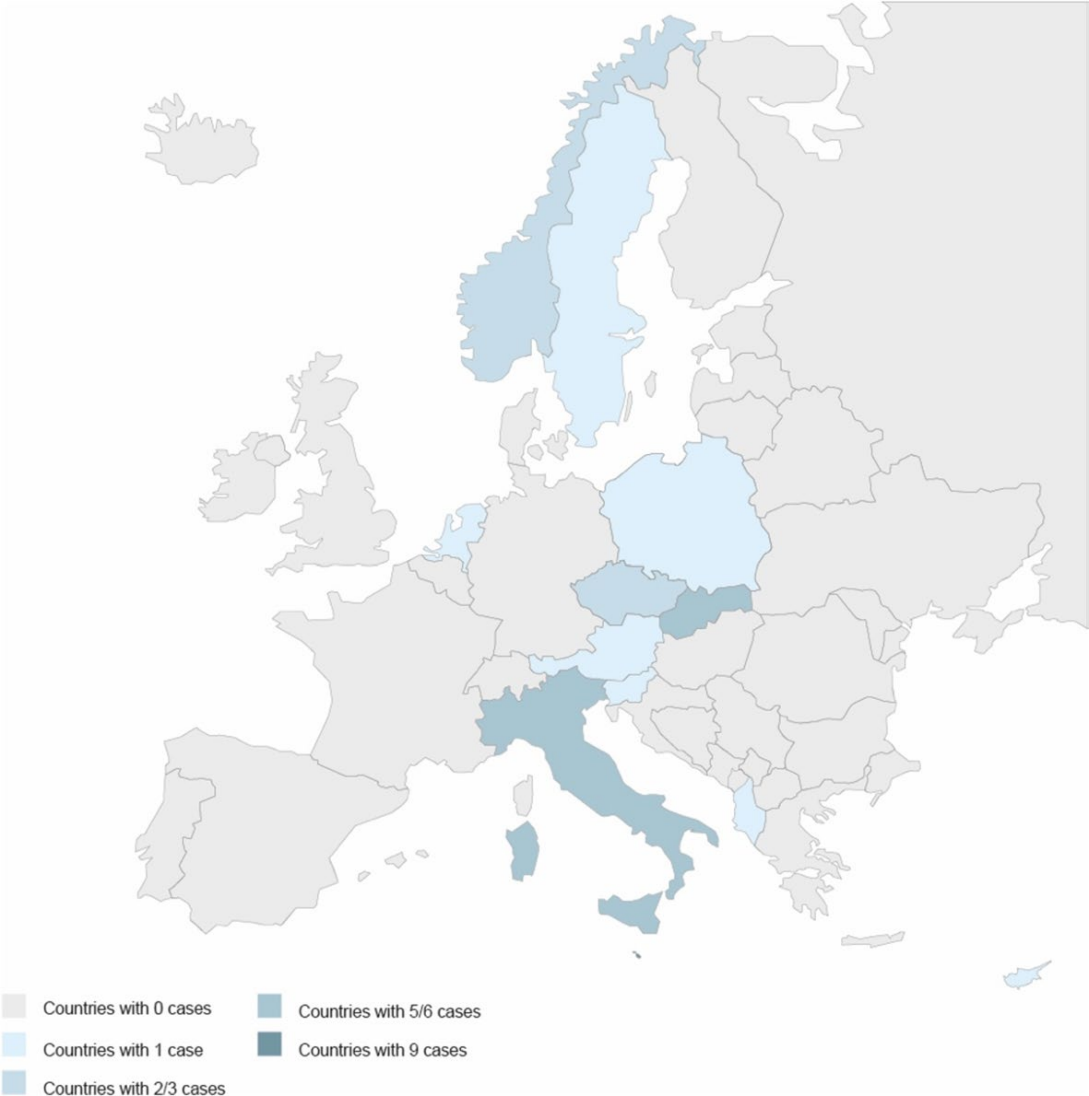
ECtHR’s cases by country. Compiled by the authors based on HUDOC data and own databases

The fact that we have no ECtHR cases on rent control before the 1980s (the beginning of the deregulation, the privatization of the housing market, and the general decrease of the social housing stock) supports the claim that the ECtHR jurisprudence aimed at facilitating the transition to a common market economy (*see Appendix*). Figure 2 shows the distribution of cases across Europe: most of the cases concern those States—such as Italy, Malta, Slovakia, and Poland—that, at the time of the decision, had strong tenants’ protections that were gradually eroded by both the national and European judiciary.

## Italy: ensuring the protection of landlords and houseowners against excessive measures

### The general framework

Unlike the ECtHR, the Italian Constitutional Court has sought to address rent controls under the Equal Protection Clause (Article 3 of the Italian Constitution) in conjunction with the Property Clause (Article 42), and with Article 41, which establishes the freedom of private economic enterprise.^26^ Over time, rent controls have sometimes been upheld and sometimes rejected by Italian constitutional judges. Once again, these cases usually arise from lawsuits in which the landlord is the plaintiff, or petitioner, and the arguments the Court makes favor the idea of a free market at the constitutional level. Italian judges also view rent control cases as balancing processes: the right to property should not be unduly restricted by rent controls and other measures to promote social welfare. Conversely, owners and landlords should not be unreasonable burdened. The arguments developed by the judges emphasize that housing is primarily a consumption good and a financial asset subtracted to legislative trade-offs that violate the right to property by imposing excessive burdens on landlords as a shortcut for ensuring (apparent) macro-economic stability in the housing market. Let us explore a few striking examples.

### Landmark decisions

In the landmark 252/1983 decision on rent control, the Court argued that “housing is a primary good protected by law, and the regulation of reasonable rent for its proper functioning is linked to the indispensable development of public and private housing […]. Housing, however, cannot be considered an indispensable condition for the inviolable rights provided for in the first part of Article 2 of the Constitution in relation to the so-called right to housing.” The Court’s reasoning went further by emphasizing that “the Constitution has not made private property a public duty but considers it a subjective right. Finally, it cannot be inferred from Article 47 of the Constitution that there is a right to housing guaranteed to all.” In sum: there is no positive constitutional right to a dwelling.

Decision 55/2000 held that rent controls do not in themselves violate Articles 3 and 42 of the Italian Constitution: the legislature is allowed to introduce exceptions to the rent control established in the Italian Civil Code. However, the rent control introduced in Campione d’Italia (a small municipality near the Swiss border) constituted a serious interference with the equality clause and the right to property, as the rent was too high in relation to market value and construction costs. The Italian legislator did not consider that the economy of Campione d’Italia is tied to Switzerland, where construction costs are much higher. This peculiarity of the real estate market was overlooked when determining the appropriate rent coefficient.

Decision 482/2000 declared partially unconstitutional Article 6(6) of Law L. 431/1998, because of the lack of a fair balance between the protection of tenants and the property rights of landlords. A moratorium on evictions from rent-controlled apartments is appropriate insofar as it provides adequate compensation to the landlord. In the same decision, the Court emphasized that rent controls should be considered as temporary measures aimed only at resolving tensions and social conflicts over housing rents in the tense and “serious” situation of the post-World War II real estate market, in support of the transition to a free housing market.

Since there are several connections between rent regulations and other tenants’ protections, and the judiciary often reasons by analogy, it would be useful to check whether the judicial behavior vis-à-vis rent regulations is consistent with a more general approach to tenancy regulations. Pursuing systematically this investigation goes far beyond the more limited scope of this essay. However, if we broaden the scope of the investigation beyond rent control cases, we find that also the recent jurisprudence of the Italian Constitutional Court on eviction confirms this trend towards ownership.

Decision 128/2021 overturned the second eviction moratorium established by “Sostegni Bis” 107/2022 for the Covid crisis. The Court found that the burden imposed on landlords was too high. The Covid 19 crisis highlighted both the impact of shock events on vulnerable residents and the constitutional relevance of landlord-tenant relations.

The Italian Constitutional Court (decision 128/2021) held that the second extension of the eviction moratorium was invalid because the sacrifice of landlords and house owners was disproportionate, also due to the lack of “selection criteria” distinguishing between tenants or reasons for arrears (*see also* 213/2021 Corte Cost. Oct. 19, 2021). This comes as no surprise: according to well-established interpretation of the Court, Article 47 “is aimed at promoting and supporting private home ownership”^27^ and not at the right to housing. This interpretation was already introduced by decision n. 217/1988, in which the Court held that a law granting low-interest loans to workers for the purchase of their first home in areas of high population growth was lawful.^28^ The Court considered this policy to implement constitutional law, as it helps those most in need to buy a housing unit. The right to housing falls under constitutional rights *framed as the right to private homeownership*.

### The relation with the Cassation Court

One might think that, in Italy, there might be a marked difference—and, indeed, a tension—between the jurisprudence of the Cassation Court and the jurisprudence of the Constitutional Court on matters of rent control. However, this is not the case: the Cassation Court has never explicitly interpreted the right to housing. Rather, the case law focuses on procedural rules for determining the reasonable price of housing and the enforcement of eviction orders.

The Cassation Court has issued several rulings on eviction proceedings involving rent-controlled dwellings. Among several decision concerning formal procedural stages,^29^ the Court established that in case of city dwellings, the evicted tenant in arrears owes both expired rental payments and the rents that will be due when the building is vacated. Even though the latter are *future debts*, they are included insofar as they constitute damages suffered by the landlord.^30^ The Italian Court of Cassation, thus, places significant emphasis on safeguarding the creditor’s interest in securing a court order against the debtor, even prior to the occurrence of non-performance by the tenant.

To conclude, the Italian judiciary, too, starting from the early 1980s, has gradually relieved the landlords from the disproportionate burden imposed by law during the post-war reconstruction phase, in a peculiar political context in which Italy had the strongest Communist Party in the Western world, which favored strong tenant’s protections. In all these cases, the primacy of the property clause (Art. 42 It Const) over the equality clause (Art. 4 It Const) means that the essential elements of the right to property—such as the right to derive economic benefit from property and the right to exclusion—cannot be sacrificed in the balancing of interests. The right to property cannot be defeated by State interventions aimed at resolving housing crises using directly privately owned assets.

## The BVerfGE: market value without maximum profit

### The general framework

The BVerfGE has decided nineteen major cases concerning rent controls between 1980 and 2021. Almost all cases were competence of the First Senate and have been assessed based solely on Art. 14 Paragraph 1 of the Basic Law (*Grundgesetz or* GG), which regulates property, inheritance, and expropriation: “(1) Property and the right of inheritance shall be guaranteed. Their content and limits shall be defined by the laws. (2) Property entails obligations. Its use shall also serve the public good. (3) Expropriation shall only be permissible for the public good. It may only be ordered by or pursuant to a law that determines the nature and extent of compensation. Such compensation shall be determined by establishing an equitable balance between the public interest and the interests of those affected. In case of a dispute concerning the amount of compensation, recourse may be had to the ordinary courts.” Only one case was addressed under the equality clause (Art. 3 Paragraph 1) read in conjunction with Art. 80a (protection of civil population under condition of tension). One can appreciate, once again, striking similarities between the BVerfGE, the Italian Constitutional Court, and the ECtHR.

The protection of the rent price and the access to a social rent (the social rent law, or “*soziales Mietrecht*”) fall under the scope of the right to property contained in Art. 14 of the Basic Law (*Grundgesetz* “GG”). According to the recent case law of the BVerfGE, the constitutional right to property protects the core elements of ownership recognized by the legal system (BVerfGE 115, 97: 110). The constitutional protection of property is therefore characterized through the “private use” and the “legal power” of the owner in regard with the object of the property (BVerfGE 31, 229: 240; BVerfGE 143, 246: Section 216). For this reason, leading scholars consider that “property” in the German Basic Law is a *cumulative concept* for the different legal statuses that convey the ownership competences of the property object (Hösch, 2000; Kempny, 2023).^31^ This constitutional right not only protects existing property rights from takings, but also ensures the owner’s exclusive availability of the asset (Sodan, 2022). This last point is fundamental because the earnings obtained from the rent of a housing unit are also constitutionally protected (BVerfGE 37, 132: 141; BVerfGE 49, 244: 247; BVerfGE 71, 230: 247). In other words, the BVerfGE declared that the constitutional protection of the property includes the so-called “substantive” property—recognized by the civil law—and the earnings derived from the lease contract (BVerfGE 79, 292).

In Germany, the right to property plays an important role for the welfare state (BVerfGE 31, 229: 240; BVerfGE 143, 246: Section 216). Thus, the right to property is regarded by legal scholars as both a private and public “defense right” against the State (Shirvani, 2016; Wendt, 2021a); this duality (private and public) becomes apparent in the regulation of the rent prices. For example, the BVerfGE has declared that there is no priority between private and public law when both regulate property (BVerfGE 58, 300: 330). Thus, the legislator has the duty to define the “content and limits” (*Inhalt und Schranken*) of property rights.

An “interference” or “breach” to the right to property obtains when the use or availability of a property is disproportionately constrained by the government. Those legal measures that set the “content and limits” to property rights (Art. 14 Paragraph 1 GG) or justify an act of expropriation through regulations (Art. 14 Paragraph 3 GG)—namely, a *regulatory taking—*might be considered as interferences with the right to property (Sodan, 2022: Section 45). The BVerfGE clearly separates these two types of interference (interference with use and regulatory takings) in its caselaw (BVerfGE 51, 1: 27–28; BVerfGE 70, 171: 199). On this note, Paragraph 2 of article 14 GG emphasizes that property “shall also serve the public good.” This clause of the GG is informed by the so-called “Social State principle” (*Sozialstaatsgebot*). In sum, the German government is bound by two conflictive constitutional duties: on the one hand, the duty to protect private property and landlord’s profits; on the other, the public interest in promoting the social inclusion of tenants and implement the welfare State (Bryde & Wallrabenstein, 2021).^32^

### Landmark decisions

The BVerfGE has developed the concept of “social accountability” (*Sozialpflichtigkeit*) in its case law to describe all the regulations that restrict the use and availability of property based on the “common good” *qua* constitutionally protected interest (BVerfGE 20, 351: Section 22).^33^ This standard for constitutional review covers rent controls, too (BVerfGE 38, 348: 370). The BVerfGE recognizes that most individuals lack the means for becoming homeowners (BVerfGE 38, 348: 370). Thus, rent regulations play a fundamental social function (which, however, shall not be regarded as an intrinsic limit of the right to property), protected by the Basic Law (BVerfGE 38, 328: 380; Shirvani, 2016). As hinted above, the restriction on property rights must respond to a principle of proportionality (BVerfGE 52, 1: 32; BVerfGE 79, 292: 302): the lawmaker can implement the duties derived from Art. 14 Paragraph 1 GG *if and only if* the landlord and tenant respective interests are *proportionally balanced* (BVerfGE 89, 1: 8). As we shall see, the balancing of the BVerfGE is essentially a trade-off between landlords’ and tenants’ competing interests (BVerfGE 37, 132: 141).^34^

Based on the case law of the BVerfGE (BVerfGE 37, 132: 142), rent controls do not yield a restriction to the right to property (Art. 14 Paragraph 1 GG) as far as the legislator ensures that landlords gain a comparative rent price (*Vergleichsmiete*) that is equivalent to the “usual” (*üblich*) housing prices within the *same* district, or a *similar* district (*Gemeinde*).^35^ Both tenants and landlords can challenge in courts a rent regulation that departs from these standards (BVerfGE 37, 132: 139; Depenheuer & Froese, 2018).

As explained above (*see above* Sect. 2), the current cap limit (*Kappungsgrenze*) of the rent price increase is 20% of the market price (since 01.09.2001, BGBl I 2001, 1149), but this can be set at 15%, when there is an actual risk of not ensuring enough housing supply (paragraph § 558 BGB). The decision of moving down to the 15% cap behooves the Federal State Governments (*Landesregierung*); in any case, the capping limit should not surpass the duration of 5 years. Specifically, the landlord’s right to increase the rent is subject to two “upper limits”. The first is the *local reference rent* (§ 558 (2) BGB) and the second is the *rent cap* itself (§ 558 (3) BGB). Meanwhile, § 558 (4) BGB specifies the cases in which the rent caps are not permitted. The lowest price of the two upper limits determines the rent increase (Wetekamp, 2023). Subsequent rent increases may not exceed 20% within a period of three years after the last increase (§ 558 (3) BGB). In principle, the rent that was paid three years before the rent increase offers the benchmark for calculating the rent cap. If the local reference rent is lower than the rent cap, the rent price may only be increased up to the local reference rent (Wetekamp, 2023).

The rent increase according to the local reference rent is regulated by §§ 558–558b BGB. The landlord can claim a rent increase based on the local reference rent if the net rent (*Nettokaltmiete*) has not been changed for 15 months (§ 558 (1) sentence 1 BGB) and the rent has not increased by more than 20% within three years. In regions with an impending housing shortage, the rent cap is set at 15%. The net (“cold”) rent is the parameter for the local reference rent.

The local reference rent is not identical to the current market rents. Rather, it corresponds to the standard installments that have been determined by the municipality (*Gemeinde*)—36 or comparable municipalities—for similar apartments. In practice, the local reference rent can be determined using a rent index (*Mietspiegel*) prepared by the municipality. The rent index consists of tables that break down the rent per m^2^ according to the type, size, furnishings, quality, and location of the apartments. The rent index is to be adjusted to market developments every two years (§ 558c, paragraph 3 BGB) in municipalities with more than 50,000 inhabitants (§ 558c, paragraph 4, sentence 2 BGB). Tenants’ and landlords’ associations can participate in the preparation of the rent index together with the municipality.^37^ Moreover, rent indices can be divided into a simple and a qualified rent indices. The first consists of the rent index prepared intuitively or through direct bargaining by the municipality (§ 558a, Paragraph 2, Number 1 and § 558d BGB), while the second type of rent index is prepared in accordance with recognized economic models endorsed by the municipality. In both cases, the rent index has two functions: *first*, it acts as a justification for the landlord’s request for a rent increase; *second*, it has an evidentiary function for reconstructing the local comparative rent and checking whether the rent increase requested by the landlord is justified (Theesfeld-Betten, 2024).

The starting point for calculating the rent cap is the time at which the rent increase takes effect (Theesfeld-Betten, 2024). The calculation involves a comparison between the planned new rent price and the previous rent price (Theesfeld-Betten, 2024).

Recently, the BVerfGE has “derogated” the general scheme, declaring that even a 30% price increase is constitutional (until 30.09.2001, BGBl I 2001, 1149) and does not yield an interference with the material content of the right to property (BVerfGE 71, 230: 250).

A “rent cap” could be considered as an interference to the right to property when its implementation leads to a disproportionate loss of profit for the landlord (BVerfGE 71, 230: 250; BVerfGE 91, 294: 310). Moreover, from the Karlsruhe Court’s perspective, the rent regulation must not burden the landlords with the risk of losing reasonable profits based on a market assessment per area (Sodan, 2022). What the Basic Law rules out, under the BVerfGE’s interpretation, is the *right to maximum profits* of the landlord (BVerfGE 71, 230: 250) and the absolute power to terminate a tenancy contract “at will” (*ad nutum*) (BVerfGE 79, 283: 289–290). Therefore, based on the BVerfGE’s precedents, any rent control that pushes the rent value under the comparative rent would be unconstitutional.

It is not entirely clear how the Court defines “comparative rent” and “maximum profit”. However, a plausible reconstruction of the BVerfGE’s model could be the following: the comparative rent is approximately the *average rental value*. Rent control regulations that set the price below the average rent would be disproportionate, as this favors tenants too much. Conversely, allowing *expected profits beyond the average rental value* (namely, “maximum profits”) would be tantamount to not intervening, which would be disproportionate, too, as it favors landlords too much. The BVerfGE therefore believes that the middle way is to take the *average rental value in the area* (*i.e*., the standard market rent) as the *fair price* for a rental unit.

Let’s assume that the average rent for a one-room apartment in a Berlin district is EUR 800/month: This means that *ceteris paribus* there are one-bedroom apartments in this district that cost, say, EUR 600/month and others that cost EUR 1200/month. The BVerfGE suggests that a reasonable (*i.e.*, “proportionate”) rent control regulation would set the price at + /– 20% of the average price, *i.e.,* 800 + /– 160 EUR/month, giving a maximum of 960 EUR/month. By saying that the Basic Law does not guarantee the right to maximum profit, the BVerfGE simultaneously suggests that the landlord is “protected” up to 960 EUR/month, which excludes the possibility of renting at 1200 EUR/month—or at any value between 960 and 1200 EUR. The sum of EUR 1200/month is, indeed, the maximum profit and could also correspond to the expected rental value in the tight market. To be sure, such a (constitutional) control measure partly protects tenants, but it may not suffice to ensure affordability in areas or cities where the market value is already too high compared to the net monthly wage, as in many European capitals. For example, if the average rent for an apartment is €1700, a ± 20% range would result in rents between €1360 and €2040. This range remains problematic for low- and middle-income households, as these prices are still beyond their financial reach. This issue is not just a theoretical case but reflects real conditions in major European cities such as Milan and Berlin. According to a recent study by Chiavazza et al. (2023), households in Milan spend an average of 43,6% of their income on rent, with some neighborhoods seeing figures that exceed 60%. In Berlin, a 2023 analysis by *21st Real Estate* indicates that residents spend an average of 32% of their income on rent, with higher percentages in central areas.^38^

It is worth emphasizing the distinction between and among new leases and existing contracts. According to the *Deutsche Bundesbank*’s “Indicator system for the German housing market”, there is a clear difference in the development of price increases between “new lettings” and “existing leases”. There has been a significant increase in new leases over the last three years. New rentals in apartment buildings have risen by 6.3% and 5.5% respectively in the cities, and by almost 7% in the seven major German cities (Deutsche Bundesbank, 2024). However, this does not mean that prices for existing contracts are not rising, but they are not rising at the same rate as new contracts. Sure enough, the general prices for existing contracts have remained stable in the period from 2019 to 2024. However, according to the Bundesbank’s data (Harmonized Consumer Prices/Germany/Source Values/04.1 Actual Rent Payments), the share of rent in the cost of living has increased significantly over the last three years. In addition, the percentage of new buyers has decreased, forcing this group to look for a rental property rather than buying a home, leading to an increase in new rentals (Deutsche Bundesbank, 2024).

Data from the *Immobilienverband Deutschland IVD* show that the cost of rents for existing apartments increased by around 53% in Germany and 86% in Berlin from 2010 to 2023, while the cost of "new" apartments (Data provided by *Bundesinstitut für Bau-, Stadt- und Raumforschung im Bundesamt für Bauwesen und Raumordnung*) increased by 153% in Berlin and 66% in Germany from 2010 to 2023; the delta for new/old apartments is 67% in Berlin and 13% in Germany as a whole.

To summarize, we can say that landlords are protected at the constitutional level in the German legal system. First, rent controls are allowed, but the judge may not rely on Art. 1 § 3 Paragraph 2 and 3 of the *Wohnraumkündigungssschutzgesetzes* to prevent the landlord from enforcing a legally permissible rent increase (BVerfGE 37, 132). Secondly, the restrictions must not place a disproportionate burden on the landlord (BVerfGE 71, 230: 250; BVerfGE 79, 80: 84; BVerfGE 91, 294: 310). Thirdly, the fair price is the market value: the right of ownership does not ensure the landlord’s maximum profit (BVerfGE 71, 230: 250; BVerfGE 100, 226: 242), which can be excluded by rent controls. However, if the rent received is lower than the average market rent, then the landlord’s economic loss is disproportionate and excessive.

### The *Mietendeckel* case

Bearing these distinctions in mind, we can now turn to the landmark Berlin’s *Mietendeckel* case, which prompted a return to discussion on the constitutionality of rent controls in Europe. In the 1980s and early 1990s, Berlin was a shrinking city. However, this trend has quickly changed in the last thirteen years: Berlin’s capital witnessed a fast economic and demographic growth. The politically driven transformation of this city into a creative and consumer metropolis has attracted knowledge capital, startups, and foreign direct investments (FDI) that enhanced economic growth.^39^ Through market regulation, urban renewal plans, tax reforms, and other incentives aimed at attracting multinational companies, footloose investments, and achieve macro-economic stability, Berlin Senate has actively promoted the socioeconomic transformation of the German capital. To be sure, this process had positive implications, such as a growth of Berlin’s GDP, higher safety levels in the city, and the increase of so-called “cafeteria effect”, determined a new migratory flux of young creatives.^40^ However, rapid urban development and economic growth comes at a cost. Due to an uptick in the demand curve, the value of housing units increases, and—with such a high demand—finding affordable housing becomes a serious issue. In the eyes of Berlin’s Senate, the possibility of introducing a rent freeze seemed a ready solution for mitigating rental property shortage at the lower-end and give a real chance of a shelter to bottom deciles.

The growing affordability crisis and the shortage of housing units—particularly in the central districts of Mitte, Prenzlauer Berg, Charlottenburg, Friedrichschein, and Kreuzberg—have severe implications, including the very possibility for low- and median- income households to accumulate wealth through property ownership. A considerable part of Berlin’s population regarded the rent freeze as a welcome State measure for contrasting high prices and real estate speculation.^41^ However, Berlin’s rent freeze had a short life: the BVerfGE held that the rent freeze was unconstitutional because it violated the vertical separation of powers as emerges from Art. 73 and 74 GG. The decision of the BVerfGE (BVerfGE 24, 367:421) was criticized by a number of academics (Ackermann, 2021; Gather & Rödl, 2021; Kummer, 2021; Steinbeis, 2021; Wihl, 2021). The main concerns were directed in particular against the use of the term “State practice” (*Staatspraxis*) for the delimitation of competences between the Federal Government and the *Länder*, and the interpretation of these constitutional competences through legal provisions (Gather & Rödl, 2021; Nispel, 2023).^42^

The Act on Rent Cap—*Gesetz zur Mietenbegrenzung im Wohnungswesen in Berlin* (MietenWoG Bln or *Mietendeckel*)—was adopted by Berlin’s Senate on February 23, 2020. This legal framework contained a set of measures aimed at protecting the economic interests of the tenants: a rent freeze that sets a “fixed” rent cap (Sections 1, 3 MietenWoG Bln); a location-independent rent cap for reletting (§§ 1,4 MietenWoG Bln); a statutory ban on excessive rents (§§ 1,5 MietenWoG Bln). The structure of Berlin’s *Mietendeckel* was very close so-called “first generation” rent control regulations introduced in Europe after WWI and WWII as emergency protections for tenants and—as hinted above—were gradually replaced starting from the 1980s with “second generation rent control measures”: by establishing a fixed cap for rent price per m^2^ in the entire city, the MietenWoG Bln reintroduced an old-fashioned regulatory approach which deviates from the prevailing approach to rent regulations in Europe that uses instead second generation rent controls and the *market value* of rental property as a benchmark for fair price.

The BVerfGE held that act was *null and void* because it violated the vertical distribution of competences between central State and *Länder*. The Court’s reasoning focuses primarily on the separation of powers between Federal State and local governments, which are regulated by Articles 73, 74, and 105 GG. These norms are front and center in determining the legislative powers of the Federation vis-à-vis the *Länder*. If the Federal Government has competing competence on a specific matter, then the normative hierarchy impedes the to endorse measures that are incompatible with the Federal law. Regulations on the matters of rent are traditionally discussed under the rubric of the tenancy law *qua* main branch of civil law, which, based on Art. 74 Paragraph 1 GG, lays within the legislative *concurrent competence* of the Federal Government. From the Court’s perspective, the German Federal Government had already exercised this competence by, *first,* promulgating Sections 556–561 of the German Civil Code (BGB) and, *second*, endorsing a national rent control scheme in 2015.

The German Federal Constitutional Court is known for basing its legal reasoning on both formalistic and substantive arguments: Proportionality and other substantive arguments are frequently used in HR cases; jurisdictional arguments, textualist interpretation and other so-called ‘formalist’ arguments are frequently used in evaluating the institutional framework and the separation of powers (Petersen, 2014; Böckenförde, 2017 pp. 152–208). However, the Court also relied on non-formalist (*i.e*., substantive reading of the constitution, purposivism, balancing, value-based reading of the constitution), including in questions of competence and vertical separation of powers (Jakab, 2013; Möllers, 2013: 13, 27 *et passim*).

In the opinion of the BVerfGE, the State practice *(Staatspraxis*) of Art. 74 para. 1, lit. 18 GG does not consider that Federal States have the competence to enact laws on the price of rent for private dwellings (*Wohnraum*).^43^ This praxis presupposes the longstanding distinction between, “publicly subsidized social housing”, “tax-privileged living space”, and “private dwellings" that has existed since the first Housing Act of April 24, 1950. This taxonomy also affects the enactment of rent regulations: also in this case, there is a strong opposition between “publicly subsidized housing" and “private dwellings”. Federal States are allowed to enact laws to regulate the rents for publicly subsidized dwellings, whereas “private dwellings” are regulated at the State level, by the German Civil Code (BGB) and Federal tenancy laws.^44^

In this sense, the BVerfGE held that the regulation of rents by the Berlin Senate went beyond the boundaries of “public housing”, stretching to the private dwelling market, which lays within the (concurrent) competences of the Federal Government. Some scholars have criticized the argument endorsed by BVerfGE. Sophia Marie Nispel (2023), for example, challenges the very idea that there is a “State practice” on matters of rent controls (*See also* Weber, 2018). Kingreen, instead, suggests that the 2006 “Federalist reform” (*Föderalismusreform*) yielded a partial devolution of competences to Berlin’s Senate, which includes the power to regulate, under exceptional circumstances, the private dwelling market (Kingreen, 2020). Finally, Hardan and Pustelnik (2021) challenge the claim that the German Civil Code and subsequent national laws on tenancy have regulated entirely the price mechanisms for the private dwelling, suggesting that *Länder* can still exert residual (concurrent) competence on pricing dynamics.

In the eyes of the BVerfGE, the *Mietendeckel* seems primarily an issue about authority and the distribution of power, not about property rights. However, this perspective is trans-substantive to some extent: as we will explain below, the distribution of powers, in this case, presupposes a conception of rent regulations as private law measures, subject to property rights constraints.

**Fig. 3:**
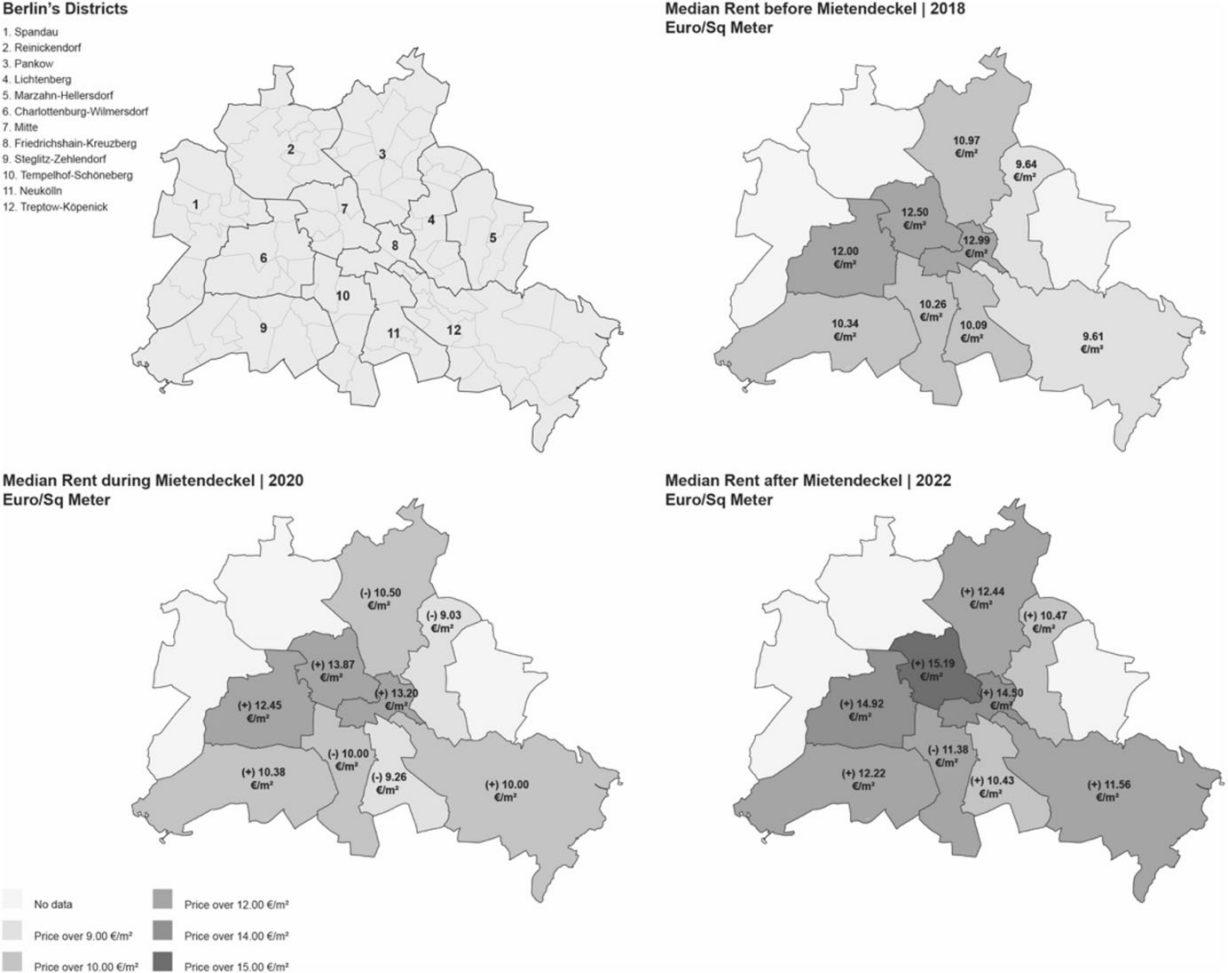
Rent increase in Berlin’s Districts. Compiled by the authors based on own calculations using data of the CBRE Group. *See Appendix f*or further details. The map shows changes in median rent before, during, and after the *Mietendeckel*

Figure 3 illustrates the dynamics of median rent prices across Berlin’s neighborhoods. Rents continued to rise apparently, the impact of the rent freeze was limited to new contracts (to determine the causal impact, a plausible counterfactual scenario should be built).

Dissatisfied with the BVerfGE’s decision, several citizens’ associations—*Berliner Mieterverein, Berliner Mieter Gemeinschaft, DGB Jugend Berlin-Brandenburg, DIDF, Gemeingut in Bürger Innenhand, GEW Berlin, IG Metall Berlin, Junge BAU Berlin, Naturfreunde Berlin, Spandauer Mieterverein für Verbraucherschutz, ver.di Berlin*—undertook a risky, disruptive, and, ultimately, unpromising approach. In particular, these organizations invoked Art. 15 of the Basic Law, which recognizes the possibility of “nationalization” (*Gesellschaftung*), in conjunction with Article 20 (1), which affirms the welfare state principle at the constitutional level. Despite its conceptual flaws, this approach brought the issue fully back to property, moving away from competence.

Art. 15 GG reads: “Land, natural resources and means of production may, for the purpose of nationalization, be transferred to public ownership or other forms of public enterprise by a law that determines the nature and extent of compensation. The Article hints at the possibility of transferring immovable property into public ownership with the aim of nationalization (Wendt, 2021b). Art. 15 mentions three objects of “socialization”. The first two objects are “land” (*Grund und Boden*) and “natural resources” (*Naturschätze*). “Land” refers to the components and appurtenances of the soil, while “natural resources” refers to the natural resources and elemental forces that are used for economic purposes (Jarass, 2022; Wendt, 2021b). Based on literal meaning, housing falls within the concept of ‘land’, insofar as the buildings are connected to and thus part of the land. Strictly speaking, private houses are not “means of production.” Rents, too, cannot be considered as part of the production process: they fall outside the extension of “means of production.” What is more, “nationalization" is a measure that the State can undertake with strong limitations, not an obligation (Jarass, 2022). Indeed, nationalization can only be performed through a formal taking provision and, according to Article 14 (3), ensuring a fair compensation.

Under a minoritarian interpretation, Art. 15 GG would allow a regulatory taking of the major real estate agencies decided by the Berlin Senate within its proper empowerments. This reading is hardly plausible. From a historical perspective, Art. 15 GG was inspired by Art. 156 GG of the Weimar Constitution, which played a key role in justifying the nationalization of the carbon and electric industry in 1918–1919. The idea of turning to Art. 15 gained currency after Berlin’s tenants’ movements promoted the referendum “Let us expropriate Deutsche Wohnen & co.!” (*Deutsche Wohnen & Co. enteignen!*), precisely proposing the socialization (*Sozialisierung*) and collectivization (*Vergesellschaftung*) of the major real estate companies operating on Berlin’s market (*Deutsche Wohnen* is the largest, hence the title of the initiative). Both a textual reading of the German Basic Law and the BverfGE precedents do not seem to support the socialization maneuver.

*First*, a strict literal interpretation of Art. 15 GG *prima facie* excludes the applicability of this norm to real estate companies: the text speaks in terms of “means of production” (*Produktionsmittel*) and, under an ordinary understanding, this term refers only to industries. Banks and insurances, for instance, fall generally outside the scope of Art. 15 GG. *A fortiori* the private housing stock.

*Second*, there is the issue related to taking compensations: the proponents of the referendum proposed a compensation based on a fair rent price (*leistbare Miete*) of 40 years, which is 25% of the current market value, which is too low. The entity of the compensation should be assessed under Art. 14 Paragraph 3 GG, which imposes an “equitable balance of interests”.

For all the above-mentioned reasons, it is hard to see how Art. 15 GG can be a viable solution. Furthermore, the notion that the housing stock plays an essential social function is only recognized by two precedents not directly connected to rent controls (BVerfGE 25, 112; BVerfGE 37, 132). However, it is difficult to see how the social connection (*Sozialbindung*) of housing could justify a restriction of the landlord’s fundamental rights to property to achieve a more symmetrical relationship with the tenants through legislative measures.

Despite these issues, a referendum actually took place on September 26, 2021. Of the 2.45 million Berliners eligible to vote, 73.5% took part in the referendum. 57.6% voted in favor of the “Expropriate Deutsche Wohnen & Co.” initiative.^45^ The result was binding, as the required quorum was reached. This meant that the Berlin Senate received plebiscitary legitimacy at the local level to take transfer part of the housing stock belonging to State-owned companies into public ownership. Whether this taking measure is constitutional is highly doubtful.^46^

## An effective protection of the right to property by the three courts

As it clearly emerges from the previous sections, starting from the 1980s, the European courts examined in this pilot study have protected property rights against strong interventions of the State on rent prices and corrected market inefficiency caused by the unintended consequences of State’s measures. The judiciary draws an essential connection between the constitutional right to property and the economic characterization of housing: *i.e*., housing belongs to the class of consumption goods and private financial assets. Accordingly, rent controls are assessed as economic relations rather than ideological or civic relations, as the activists of the *Mietendeckel* case desire: lowering the rent below market value and reasonable return on investment is unacceptable (*Hutta-Czaspka* § 40), as it interferes with the core of ownership rights.

In particular, Iannello’s (2022) shows the unintended consequences of rent controls in Italy. Rent control in Italy was primarily aimed at stabilizing the housing market and curbing inflation. However, this policy had several unintended economic consequences: housing market distortions, reduced housing quality, and decreased mobility. Controlled rents were kept artificially low, leading to significant disparities between controlled and uncontrolled rents.

For example, in the early 1960s, controlled rents were often less than half of uncontrolled rents, with controlled rents averaging around 2846 lire compared to 6879 lire for uncontrolled rents in the largest cities (populations over 1 million).

The low rent levels provided little incentive for landlords to maintain properties, leading to a deterioration in housing quality and a reduction in available rental housing. The disparity between controlled and uncontrolled rents created strong incentives for tenants to remain in controlled properties, reducing overall residential mobility.

Iannello’s study is particularly notable for its reconstruction of detailed rent data from the Istat historical archive, which allowed for a nuanced analysis of the rent control impacts across different periods and city sizes. From 1947 to 1975, controlled rents increased modestly due to periodic legal adjustments, while uncontrolled rents rose more sharply. For instance, from 1953 to 1963, controlled rents increased from an average of 2846 to 4798 lire, whereas uncontrolled rents went from 6879 to 8243 lire in large cities. The gap between controlled and uncontrolled rents varied by city size.

In cities with populations over 1 million, the gap was around 4032 lire before the 1963 rent freeze, narrowing to 3444 lire afterwards. In smaller cities (under 100,000 inhabitants), the gap was significantly smaller, around 2115 lire before the freeze and 738 lire after.

The study highlights that rent control was used strategically to manage inflation and labor costs. During periods of high inflation, such as post-WWII and the 1970s, strict rent controls were imposed to prevent a rise in labor costs that could fuel further inflation. For example, after the 1969 wage shock, a new rent freeze was implemented to curb inflationary pressures.

Iannello’s analysis underscores that Italian rent control policies, while ostensibly aimed at tenant protection, primarily served broader economic objectives. By stabilizing rents, the government managed to keep inflation and labor costs in check, essential for maintaining Italy’s competitive edge in international markets during its post-war economic boom.

**Fig. 4:**
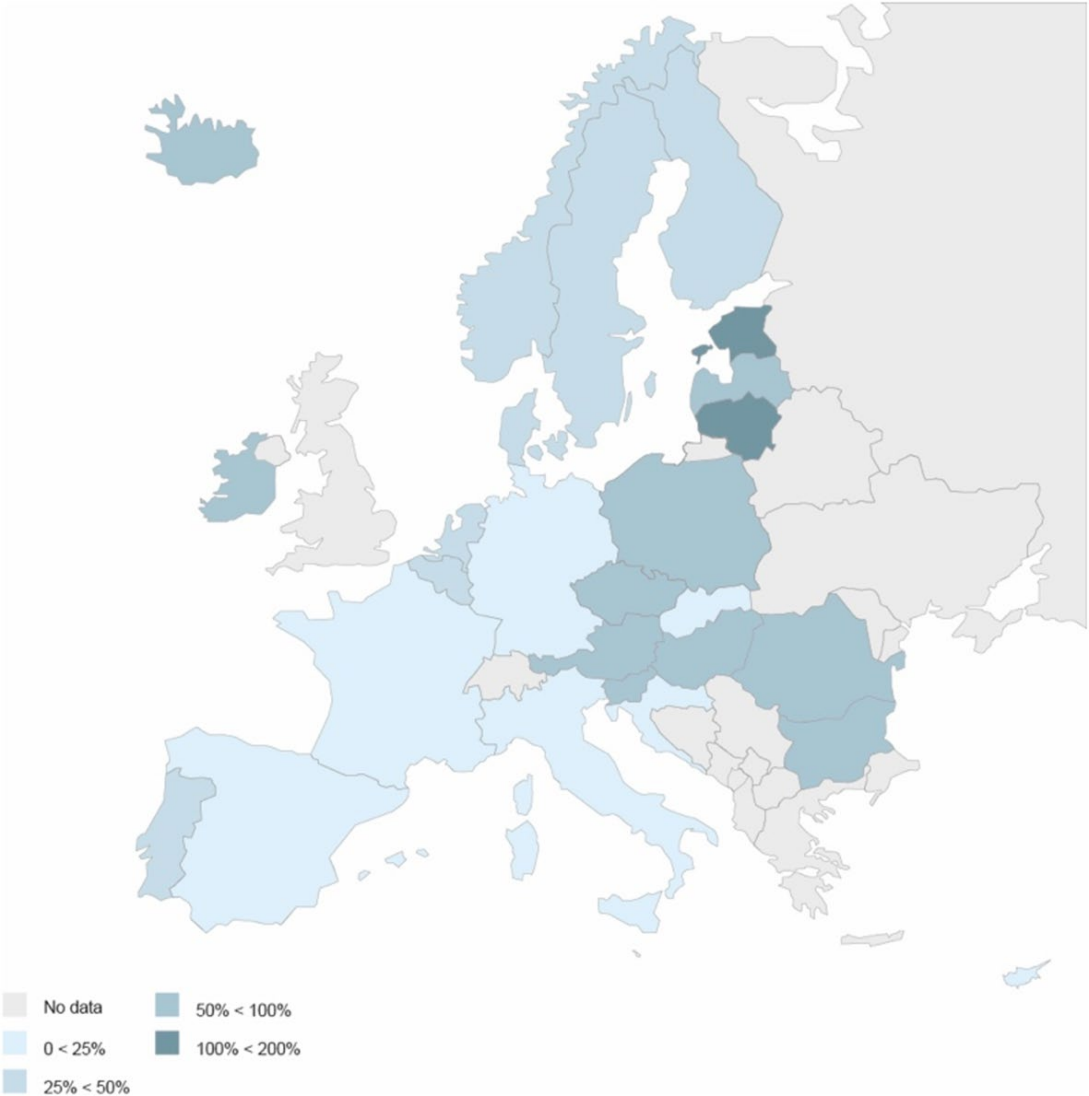
Increase of average rent prices in Europe between 2010 and 2023. Authors’ own elaboration based on data taken from EUROSTAT, 2,023

Even in a context of increasing rent prices and housing crisis, such as that of the EU housing market (*see* Fig. 4), the economic dimension of ownership takes precedence over the State’s interest in using directly private housing stock as a primary asset for redistribution. This trend is present even in the Italian Constitutional Court, which seems most protective of tenants compared to the others. Affordable housing must be provided *directly* by the State, without using *indirectly* the housing assets of individuals and private companies. The rental price, instead, must be the result of a symmetrical negotiation between the contracting parties. The Courts recognize in principle that national governments have a legitimate interest in protecting vulnerable tenants from unaffordable housing. However, the right to affordable housing does not have a *horizontal effect* that justifies rent controls below market value or that cannot be considered an ephemeral measure, being otherwise an “implicit waiver” of property or, at best, a form of unjust enrichment.

In all the cases analyzed, the judiciary treats the right to property as a side-constraint to (horizontal) social justice: property is still the most important right that usually takes precedence in case of conflict (Sacco, 1968). However, to achieve a proportional balance between protection of ownership and a national interest to affordable housing, the judiciary rules out the landlord’s entitlement to a *maximum profit* from the scope of the right to property. This reasoning can also be applied to cases involving security of tenure: protection against eviction is primarily procedural and not substantive. An economic conception of property as the right to undisturbed use of possession and profit clearly emerges from the case law.

Sure enough**,** this was not the only paradigm of property in Europe, particularly before the 1980s. At that time, a socialist theory of property was also circulating. Following the corporativist movements of the XIX century, the Weimar Constitution (August 11, 1919) Art. 153 Par. III, for example, explicitly recognized the so-called essential social function of property: “Property obliges. Its use shall at the same time be service for the common good.” (*Eigentum verpflichtet. Sein Gebrauch soll zugleich Dienst sein für das Gemeine Beste*) (Tarello, 2024). Socialist notions of property are now anachronistic in EU democracies (Berger Krall & Others §§ 120–1). *Pace* Calabresi, there is no room for considering housing as a *merit good* (Calabresi, 2016), to wit: a good whose allocation mechanism is subtracted from the prevailing market and price dynamics simply because the prevailing market allocation would generate “moral costs.”

The best explanation of the Court’s characterization of property rights—as it emerges from the case law—is given by the economic theory of the right to property, first elaborated by Alchian and Demsetz (1973: 17ff). The right to property has a core that cannot be subject to trade-off and sacrificed vis-à-vis States’ redistributive interests. This claim has been consistently endorsed by the Italian Constitutional Court, the BVerfGE, and the ECtHR.

In the economic theory of rights, the right to property is conceptualized as comprising several core elements that are essential for ensuring the efficient allocation and protection of resources. These core elements are three.

*First*, the *Ius Excludendi* (Right to Exclude), which allows the property owner to prevent others from using or interfering with their property. The right to exclude is critical for maintaining the value and utility of the property, as it secures the owner’s control and reduces potential conflicts over resource use (Alchian & Demsetz, 1973: 17ff.).

*Second*, the *Ius Fructus* (Right to Fruits). This element refers to the right to derive economic benefits from the property, such as rents and profits. The right to fruits creates incentives for investment and optimal resource utilization, as property owners can internalize the returns on their investments. This aligns with the principle of maximizing the net present value of property-related cash flows (Posner, 1973: 30ff.).

*Third*, the *Ius Abutendi* (Right to Use and Abuse), which includes the right to use the property according to the owner’s preferences, including making modifications or even destroying it. The right to *abutendi* ensures that property can be employed reflecting individual utility functions and preferences that coalesce into particular bundles (Demsetz, 1967: 347 *et passim*).

These three core elements collectively ensure that property rights promote economic efficiency and individual welfare by providing clear entitlements and reducing transaction costs associated with resource allocation and protection. The effective enforcement of these rights is crucial for reducing hold-up problems, mitigating the tragedy of the commons, and facilitating Coasean bargaining (Coase, 1960).

As we have seen from the extensive analysis of the case law, the three Courts explored in this pilot study apply consistently and effectively this model for the protection of property against rent controls at the HR and constitutional level: if rent controls undermine the right to fruits and the right to use of the landlord, then they are unconstitutional; if they are mechanism aimed at establishing the (fair) market price, and avoid speculations in tight housing markets, then they are legitimate. In both cases, States must respect a general principle of competence.

## Context analysis

We are still owed with an answer on a fundamental question: what are the macro-economic trends underpinning the expansion of the jurisprudence on rent control after the 1980s and the continuous promulgation of new rent control regulations in the EU?

There is a clear indication of a rising interest in reinstating rent controls following the 2008 economic and financial crisis. Furthermore, the considerable inflation rates and urban renewal processes in recent years have undeniably contributed to fuelling this interest. What is more, the weaker economic condition of most tenants—determined by trends in labour markets and workers’ income—have put pressure on both States and Courts.

We believe that the shifts in the tenants’ economic condition should be viewed comprehensively, considering the evolutions in society, family dynamics, the economy, and, more specifically, the trends in the labour market and workers’ incomes. The rental contract is, both in the Italian and German cases, a widespread residential agreement among less affluent families compared to more affluent ones. Taking the Italian case as an example, among the less affluent, approximately 32% of households are in rental arrangements (*i.e.*, 1 in 3 households), while among the more affluent, it drops to around 11% (Istat, 2022).^47^

This brief contextual analysis aims to highlight, with supporting data, the progressive weakening of the working class and, more broadly, the lower-middle class (and, *de facto*, most tenants) in recent decades, underscoring the need, in this historical phase, to rethink the measures for contrasting marginalization in the housing market. It is likely that, in this situation, the combined effect of various factors—that we will try to briefly summarize hereafter—makes the burden on tenants unsustainable. The States turned to rent controls to put a patch on this pressing social issue, instead of tackling with the complexity of the housing markets dynamics. HR and Constitutional Courts responded, by protecting the core elements of the right to property against disproportionate State actions.

### Labour market reforms

In Italy, over the past three decades, there have been significant labour market reforms, which should be viewed within the framework of a European strategy aimed at achieving the so-called “flexicurity”. The goal of this strategy was to simultaneously ensure both flexibility (in hiring and firing)—eagerly sought by businesses—and worker security. Initial steps in this labour market reform process included the Treu Package (Law 196/1997) and the Biagi Law (Law 30/2003), which increased flexibility in hiring through atypical and precarious contractual forms. Subsequent measures, such as the Fornero Law (Law 92/2012) and the Jobs Act (Law 183/2014), focused on increasing flexibility in terminations.

**Fig. 5:**
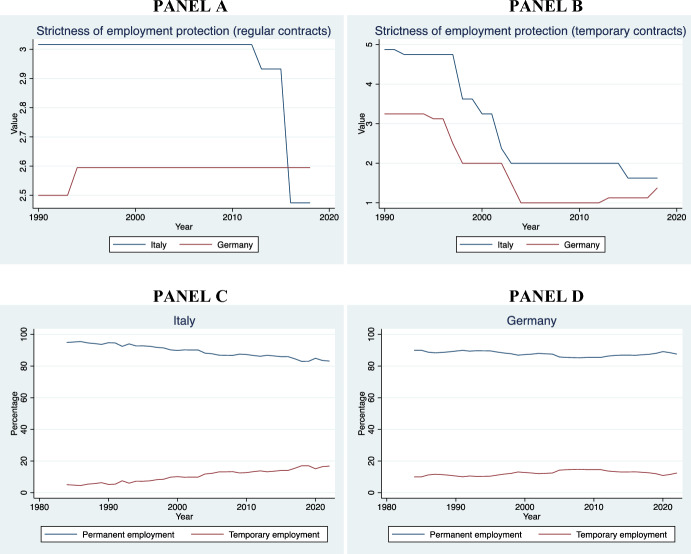
Labour market trends in Italy and Germany. Compiled by the authors. The data source is OECD. In the case of regular contracts, the strictness of employment protection refers to individual and collective dismissals

Figure 5 provides summary information on the trends of two important labour market variables from 1990 to 2018: the employment protection legislation (EPL) and the percentage of temporary and permanent workers within the total workforce.

A reduction in employment protection legislation is evident for both regular contracts (Fig. 5, Panel A) and temporary contracts (Fig. 5, Panel B), along with the progressive increase in temporary workers and the corresponding decrease in permanent workers (Fig. 5, Panel C). According to the INAPP 2022 Report,^48^ among the new contracts activated in 2021, only 15% were permanent contracts, with more than 8 out of 10 contracts being atypical employment contracts.^49^

Over the past thirty years, Germany has undergone labour market reforms, albeit to a lesser extent and in a different manner compared to Italy. Nevertheless, these reforms have significantly impacted employment security and the broader working conditions of individuals. Notably, the introduction of Mini-jobs in 2003 and the Hartz reforms (2003–2005) enhanced the flexibility of indefinite-term contracts, followed by a partial reversal in 2017 aimed at improving employment security.

Figure 5 illustrates that, unlike the Italian scenario, the reduction in employment protection did not occur concerning regular contracts—for which it even increased—(Panel A) but only in relation to temporary contracts (Panel B). Regarding the distribution between permanent and temporary workers, although there has been a slight increase in temporary workers and a minor decrease in permanent workers over the three decades (Fig. 5, Panel D), these shifts are much less pronounced compared to the Italian case.

As a complement to this concise overview of the labour market’s evolution in the last 30 years, it is noteworthy to mention the dynamics of real wages. According to OECD data (2023),^50^ real wages in Italy decreased by 2.9% between 1990 and 2020, while in Germany, they increased by 33.70% between 1991 and 2020. Furthermore, it should be noted that while both Italy and Germany have developed a system of collective bargaining regarding wages and working conditions (albeit heterogeneous among themselves), to date, only Germany has a national minimum wage.

### Inequality trends

Beyond the changes in the labour market, the contextual situation, and several other factors, including the multiple crises that have occurred in recent years (currency crises, economic-financial crises, the pandemic, and the return of war in Europe), have led to a reversal in the dynamics of income inequality, resulting in a reduction in the middle class and workers’ purchasing power.

While income inequalities in Italy decreased from the 1960s to the late 1980s, Fig. 6 (Panel A) highlights a consistent decline in the share of pre-tax national income held by the bottom 50% (*i.e.*, the 50% of the population with the lowest incomes), dropping from around 21% in 1984 to 15% in 2022. In contrast, the share held by the top 10% (as well as the top 1%) has consistently increased: the top 10% of the population held 35% of the pre-tax national income in 1984, rising to 39% in 2022, while the top 1% increased from around 9.5% to 13.5% over the same period.

**Fig. 6:**
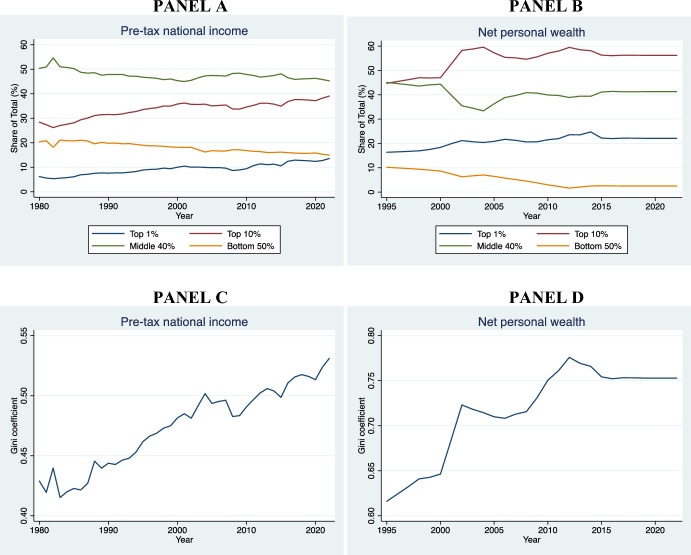
Income and wealth inequality trends in Italy. Compiled by the authors. The data source is the World Inequality Database (WID.world). In Panels A and C, income is measured before considering taxes and transfers. In Panels B and D, net wealth of households is the sum of financial and non-financial assets owned by individuals, net of their debts

By 2022, the top 1% is close to having the same pre-tax national income as the bottom 50%. The data refer to incomes before the redistributive intervention of the State, which, however, has not succeeded in mitigating these differences. A similar trend is evident in relation to net personal wealth (*see* Fig. 6, Panel B): the wealth held by the top 10% of the population has increased from 44.5% in 1995 to 56% in 2022, and that of the top 1% has increased from 16 to 22% in the same period. In contrast, the wealth held by the bottom 50% has decreased from 10 to 2.5%.

The same dynamic is depicted by the increasing trend of the Gini index,^51^ one of the most utilized by economists, both concerning pre-tax national income (Fig. 2, Panel C) and net personal wealth (Panel D).

**Fig. 7:**
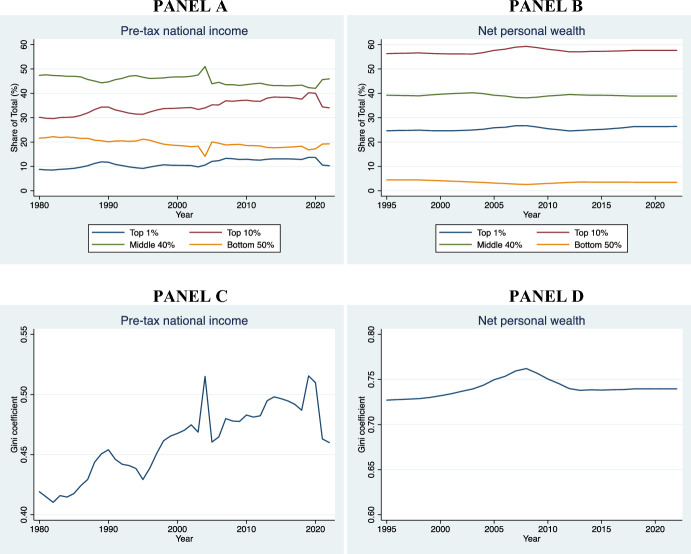
Income and wealth inequality trends in Germany. Compiled by the authors. The data source is the World Inequality Database (WID.world). In Panels A and C, income is measured before considering taxes and transfers. In Panels B and D, net wealth of households is the sum of financial and non-financial assets owned by individuals, net of their debts

The dynamics of income and wealth inequality in Germany differ from those in Italy (Fig. 7): although income inequality has increased, as indicated by the rise in the Gini coefficient of pre-tax national income (Panel C), the increases in the income share held by the top 1% and 10% of the population have grown much less compared to the Italian case. Additionally, the share held by the bottom 50% has decreased slightly (from around 21.5% in 1980 to approximately 19.5% in 2022—*see* Panel A). The same can be observed regarding wealth distribution (*see* Panel B and Panel D). However, while the situation has worsened to a lesser extent over the past three decades compared to the Italian case, even in Germany, the bottom 50% has less than 20% of the pre-tax national income and approximately 3.5% of the net personal wealth.

### The uneven burden of inflation

**Fig. 8:**
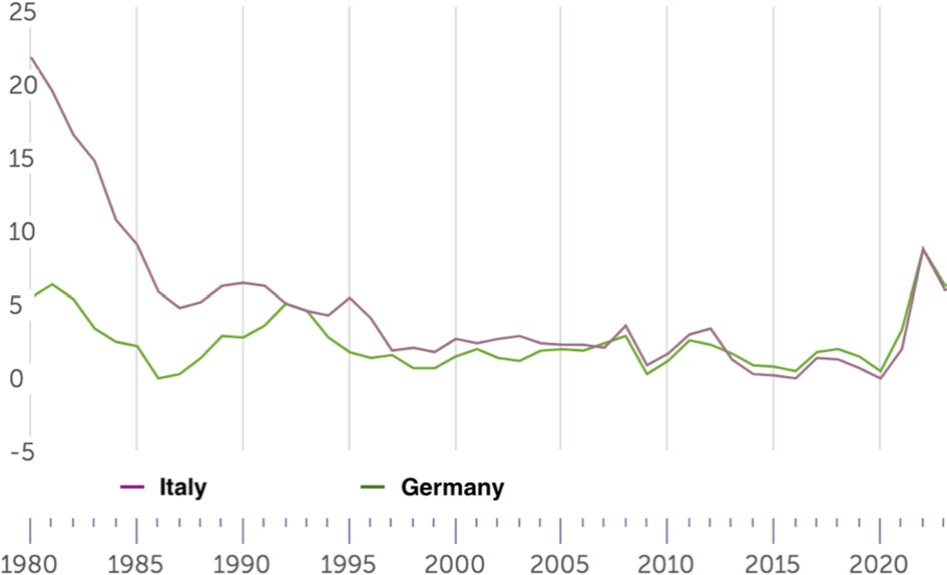
Inflation rate in Italy and Germany (1980–2023). The chart is included in the World Economic Outlook (2023) by the International Monetary Fund (IMF). For the calculation of the inflation rate, the percent change in the average Consumer Price Index (CPI) is considered

The analysed data highlight that, on average, workers are facing reduced protection, lower salaries, and diminishing wealth. Therefore, it is reasonable to assume that they have already experienced a decline in purchasing power over the past decades, further exacerbated by the recent double-digit inflation. Figure 8 illustrates the inflation rates over the last forty years in Italy and Germany, revealing that both countries experienced inflation rates in 2022 and 2023 that had not been seen in over thirty years. Additionally, during the 1980s, characterized by high inflation, the percentage of inflation-indexed employment contracts compared to the total number of employment contracts was much higher than it is today in both Italy and Germany.

It is worth noting that, concurrently with the rise in inflation, obtaining a mortgage has become more burdensome (*i.e.*, interest rates have increased) and rental costs have risen.

While it is widely accepted among economists that high inflation rates disproportionately impact low incomes compared to high incomes,^52^ it is also true that the burden of inflation on different income groups is not uniform across all States. Claeys et al. (2022) have highlighted that this discrepancy is primarily due to more similar consumption patterns among high and low-income households in some countries than in others, the greater increase in prices of goods predominantly consumed by high-income households in certain States rather than others, and the implementation of different national policies by various countries.

**Fig. 9:**
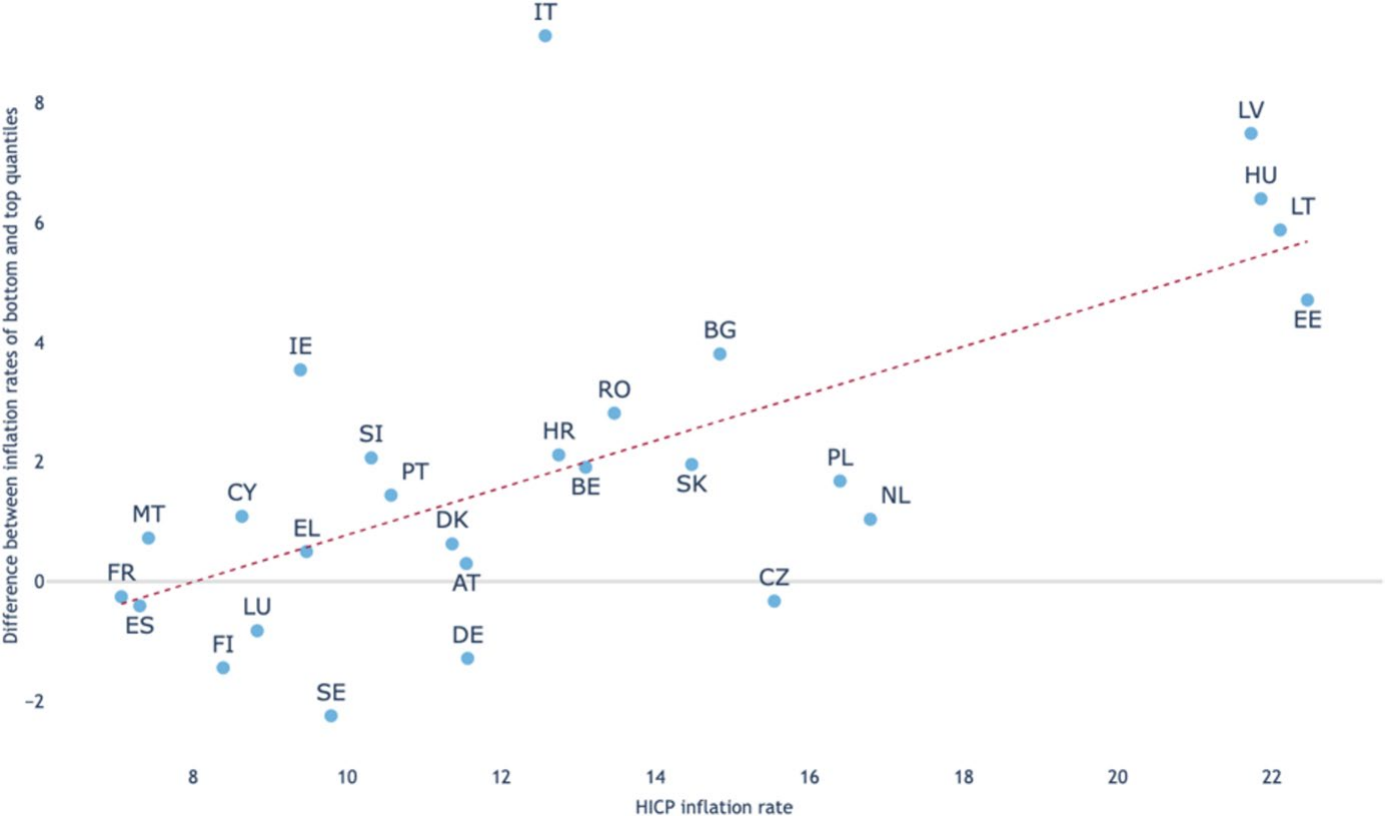
Inflation rates and inflation inequality—EU 27 (2022). The graph was created by the Bruegel Institute using data from Eurostat and national statistical institutes. The data refers to October 2022. The Y-axis represents an indicator of the perceived inflation differential between the bottom and top quintiles of the income distribution. The X-axis depicts the inflation rate. Specifically, the acronym HICP stands for the Harmonized Index of Consumer Prices—an index used to measure inflation consistently when considering different countries

Figure 9 illustrates that Italy and Germany, despite having similar inflation rates (*see* Fig. 8, period 2020–2023), show a discrepancy in the inflation rates experienced by the bottom and top quantiles of the income distribution.

**Fig. 10:**
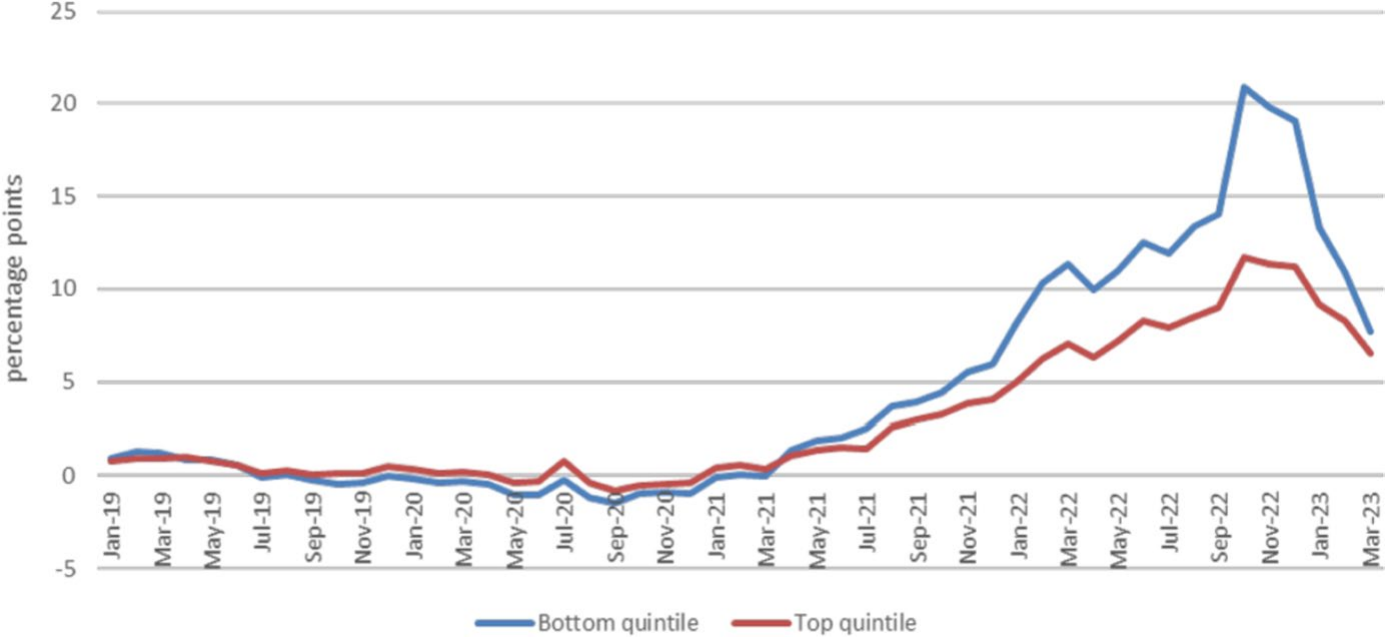
Inflation inequality—Italy. The graph was prepared by the authors of “Inflation and Inequality”—Monetary Dialogue Paper, June 2023 (European Parliament), using data from the Bruegel Institute. For further information, refer to the mentioned document

**Fig. 11:**
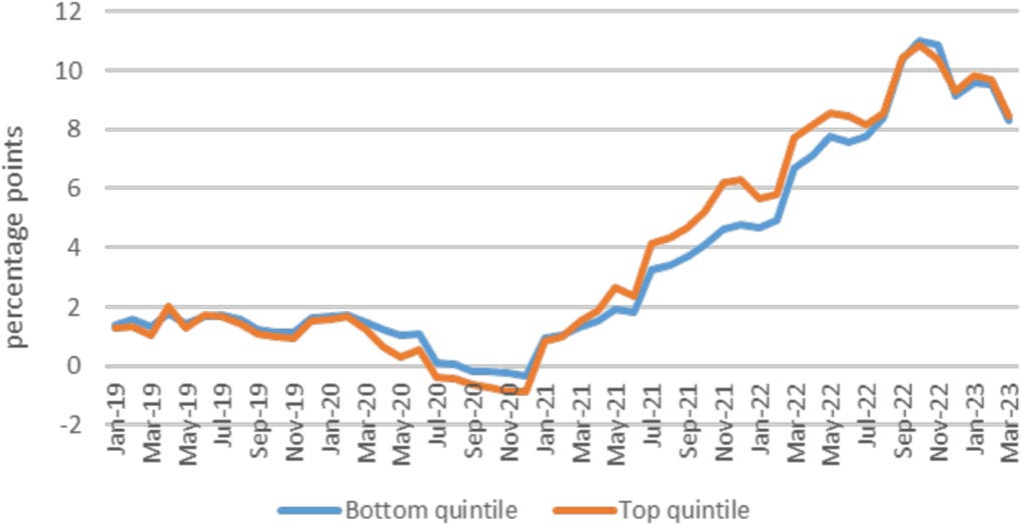
Inflation inequality—Germany. The graph was prepared by the authors of “Inflation and Inequality”—Monetary Dialogue Paper, June 2023 (European Parliament), using data from the Bruegel Institute. For further information, refer to the mentioned document

A comprehensive analysis of Fig. 9, 10, and 11 highlights that the burden of inflation has been more evenly distributed among the more and less affluent in Germany compared to the Italian scenario. In Italy, the bottom quintile (*i.e.*, the lowest 20% of the population by income) experienced a higher inflation rate than the top quintile between 2021 and 2023 (*see* Fig. 10). Conversely, during the same time frame, the inflation rates perceived by the bottom and top quintiles in Germany were much more similar and, for certain periods, even higher for the top quintile (specifically, the period from 2021 to 2023, *see* Fig. 11).

### Household trends

**Fig. 12:**
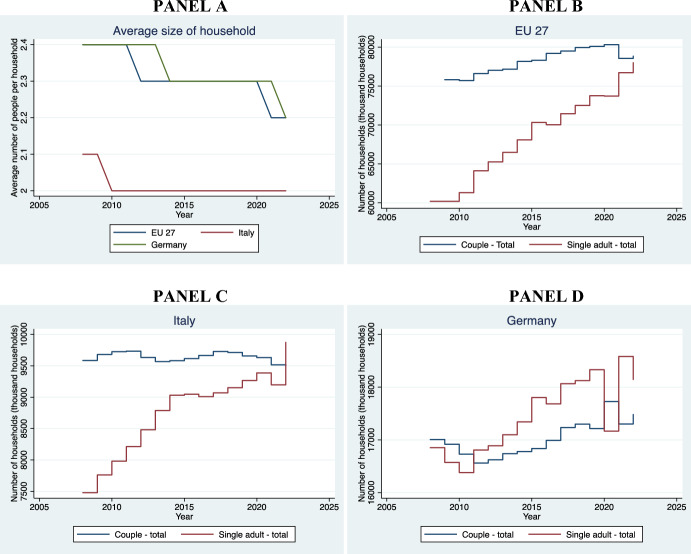
Households composition in EU 27, Italy and Germany. Authors’ elaboration based on Eurostat data (2023)

Furthermore, against the backdrop of these economic shifts, family structures are undergoing changes. Figure 12 illustrates how, over the last 15 years, the average size of households has gradually decreased both at the aggregate level (EU 27) and in Germany and Italy (Fig. 12, Panel A). Similarly, there was an increase in the number of single-person households, a trend observed in both the EU 27, Italy, and Germany (*see* Fig. 12, Panel B, C, and D). According to Eurostat (2023),^53^ the number of single-person households without children in the EU increased by approximately 31% between 2009 and 2022. This phenomenon poses a more significant challenge for Italy, characterized by a familistic welfare regime, compared to Germany, which follows a corporatist welfare system (Arbaci, 2019). Italy, emphasizing the family’s role as an economic and social support, appears less equipped to provide social assistance and support in the face of this progressive shift and reduction in the average size of families.

The rate of households in rental properties among the more affluent stands at approximately 1 in 10 (Istat, 2022). Therefore, it is reasonable to assume that most landlords belong to the middle and lower-middle class.

### Housing affordability

In this context, the cost of housing has become increasingly significant. In recent years, housing affordability has emerged as a central issue in public discourse. Over the past 30 years, housing expenditure as a share of final consumption expenditure has consistently increased in both OECD countries and EU member states, reaching levels considerably higher than those of the 1990s.

**Fig. 13:**
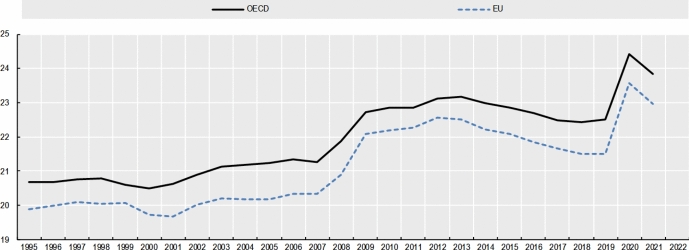
Housing expenditure as share of final consumption expenditure of households (1995–2021). The definition of housing expenditure used here is extensive and includes housing, water, electricity, gas, and other fuels. Specifically, the graph shows housing expenditure as a share of final consumption expenditure of households (OECD average and EU average), 1995–2021. The data and the graph are sourced from the OECD Affordable Housing Database, specifically the report “Housing-related expenditure of households”. For further details, see: OECD Affordable Housing Database

Figure 13 illustrates this trend, although it represents an average across many countries and includes an extended definition of housing costs that encompasses not only housing expenses but also utilities like electricity, gas, and other fuels. Additionally, it does not differentiate between homeowners and renters, providing only a general trend without detailed insights into specific developments. Thus, a deeper analysis of housing affordability, particularly focusing on Europe, Germany, and Italy, is necessary.

**Fig. 14:**
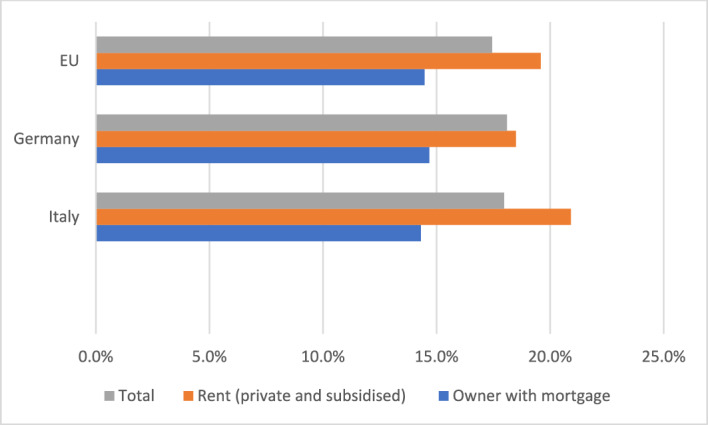
Households’ housing cost burden (mortgage and rent cost) as a share of disposable income (in %, 2022). The graph shows the median of the mortgage burden (principal repayment and interest payments) or rent burden (private market and subsidised rent) as a share of disposable income, in percent, 2022 or the latest year available. The data and the graph are sourced from the OECD Affordable Housing Database, specifically the report “Housing costs over income”. For further details, see: OECD Affordable Housing Database

Figure 14 details the housing cost burden (mortgage and rent) as a percentage of disposable income for the European Union, Germany, and Italy, distinguishing between renters (both private and subsidized) and homeowners with a mortgage. The data reveal that renters allocate a larger share of their disposable income to housing costs compared to homeowners across these regions. Specifically, the OECD data in Fig. 14 shows that renters’ housing cost burden relative to disposable income exceeds that of homeowners with a mortgage by 3.8% in Germany, 6.6% in Italy, and 5.1% on average across the EU. This indicates that renting households face a higher proportional housing cost. Moreover, these figures likely understate the true burden for renting households, as subsidized rents, included in the OECD data, lower the average cost. The burden would be higher if only private rental data were considered.

These data, however, conceal significant heterogeneity at the sub-national level and consequently underestimate the situations that require closer attention. Nationally, the data indicate that both rent and mortgage burdens on disposable income are below 30%—the commonly accepted threshold above which housing is not considered affordable.^54^ However, this threshold is significantly exceeded in many major cities, such as Berlin and Milan, where rent costs have reached very high percentages of income. Similar patterns are observed in various provinces and regions when examining lower income brackets or younger age groups, rather than average or median income.

According to a 2021 survey by Idealista, Italy’s leading real estate portal, households in Milan face an average rent cost equal to 42.6% of their income.^55^ A recent study by Chiavazza et al. (2023) reports a similar Fig. (43.6%) and highlights that, in some neighborhoods of Milan, this ratio exceeds 60%, double the threshold of affordability.

**Fig. 15:**
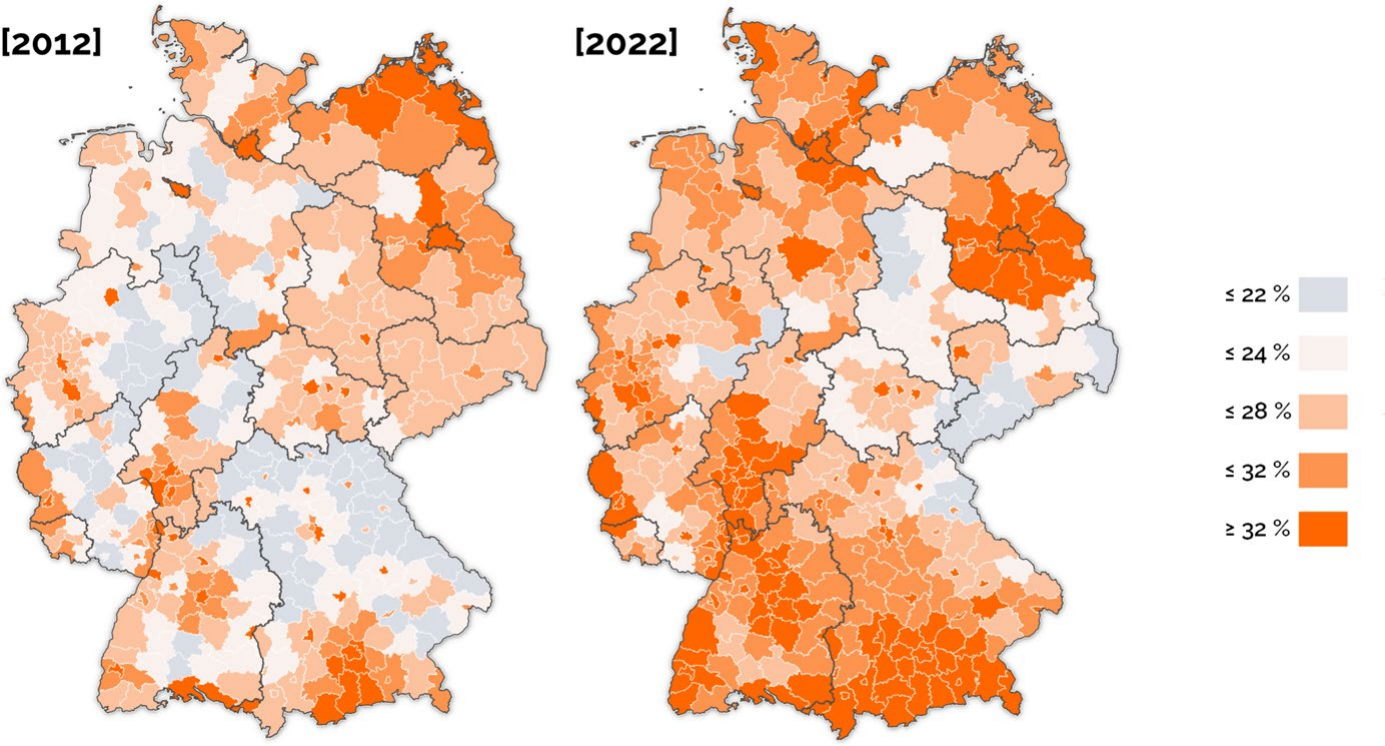
Rent burden with an income in the lower third of households (all households) for a 60–80 new lease apartment. https://www.empirica-regio.de/en/blog/240124_rent_burden/

In Berlin, a 2023 analysis by 21st Real Estate indicates that residents spend an average of 32% of their income on rent, with higher percentages in central areas. Figure 15 further illustrates that in many German regions, the rent burden for the lower third of households exceeds 32%, a marked increase from ten years ago. This issue extends beyond major cities and affects lower income brackets more broadly.

In this context, it is important to note that the share of homeowners varies significantly across different income levels. Table 1 shows that the proportion of homeowners in the top income quintile is 36.3% higher in Germany, 26.8% higher in Italy, and 26.1% higher across the EU compared to the bottom quintile.

**Table 1:** Share of homeowners across the income distribution

	Bottom quintile (%)	2nd quintile (%)	3rd Quintile (%)	4th quintile (%)	Top quintile (%)
Germany	24.9	33.1	40.9	50.7	61.2
EU	**59.5**	**67.8**	**75.0**	**80.6**	**85.6**
Italy	61.6	66.2	73.3	78.9	88.4

Bold value indicates the percentages

The data are sourced from the OECD Affordable Housing Database, specifically the report “Housing costs over income”. For further details, see: OECD Affordable Housing Database

Low-income households, which are predominantly renters and have lower incomes, are disproportionately affected by rising rent costs. The OECD Affordable Housing Database offers additional insight by showing the share of low-income private tenants spending more than 40% of their income on rent, exceeding the 30% threshold by 10 percentage points (data updated to 2022). Specifically, this proportion is 16% in Germany and 28% in Italy, with figures likely higher in major cities such as Milan and Berlin.

The OECD Risks That Matter Survey (2022) provides a measure of perceived housing affordability. It assesses the proportion of respondents (both owners and tenants) who are “concerned” or “very concerned” about their ability to find or maintain adequate housing. In both Italy and Germany, 48% of respondents reported being “concerned” or “very concerned”, indicating a significant level of concern.

Table 2 reveals that this concern is even more pronounced among certain groups, including tenants (67% in Italy, 59% in Germany, and 64% on average across the OECD), individuals in the bottom income quintile (67% in Italy, 62% in Germany, and 59% on average across the OECD), and those aged 18–24 (64% in Italy, 72% in Germany, and 61% on average across the OECD).

**Table 2:** Share of people concerned about being able to find/maintain adequate housing by tenure, income quintile and age

	Tenant (%)	Owner (%)	Bottom quintile (%)	Third quintile (%)	Top quintile (%)	18–24 years old (%)	25–64 years old (%)
Italy	67	40	67	39	41	64	45
Germany	59	31	62	45	37	72	45
OECD-27	**64**	**39**	**59**	**47**	**39**	**61**	**46**

Bold value indicates the percentages

The data are sourced from the OECD Risks That Matter Survey (2022), specifically from the report “Subjective Measures on Housing.” The survey question was: “Thinking about the next year or two, how concerned are you about not being able to find or maintain adequate housing?” Respondents could choose from the following options: (1) Not at all concerned; (2) Not so concerned; (3) Somewhat concerned; (4) Very concerned; (5) Can’t choose/Not applicable. The table displays the percentage of respondents who selected either “Somewhat concerned” (option 3) or “Very concerned” (option 4)

Overall, these data underscore the decline in housing affordability over recent decades and highlight it as a critical issue, particularly for low-income families who are also contending with high inflation, rising energy costs, and stagnant wages. Addressing this challenge has become one of the foremost priorities for policymakers.

## Summary

From this brief contextual analysis, it is evident that over the last 30–40 years, the condition of the bottom 50% of the population in Italy has consistently worsened, both in terms of pre-tax national income and net personal wealth. During the same period, most workers, whether permanent or temporary, have experienced a reduction in employment protection levels, and businesses have predominantly made new hires through temporary contracts.

In this scenario, the weight of inflation—particularly high in recent years—has ended up bearing more heavily on the less affluent, especially in Italy. Meanwhile, the size of families continues to decrease, and the number of single-member households is on the rise. This factor is not without consequences, especially in a nation like Italy, characterized by a familistic welfare regime where the family serves as the primary means of social support and assistance. These dynamics closely concern tenants, as less wealthy households are typically in this category.

Furthermore, it is worth noting that the significant growth of Airbnb as a tool available to individuals for short-term rentals has contributed to the decrease in the number of medium to long-term rental properties, leading to increased costs in various cities.

**Fig. 16:**
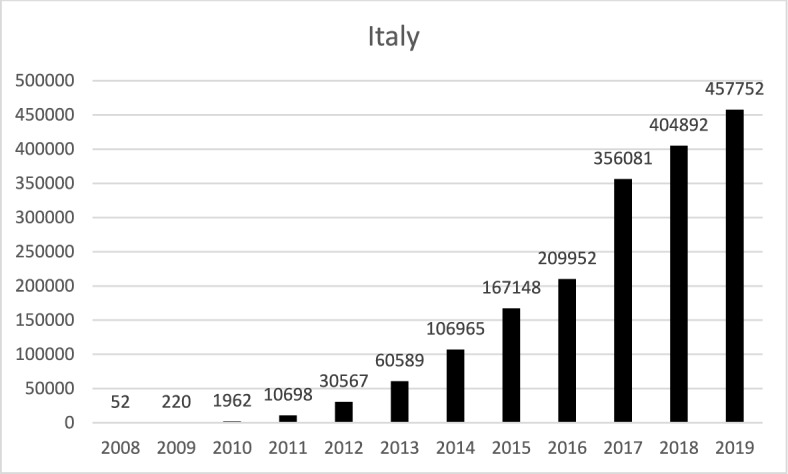
Number of accommodation listings on Airbnb in Italy (2008–2019). Authors’ elaboration based on Statista data from Inside Airbnb surveyed by INCIPI Consulting Società Cooperativa, Survey name “Tourism and shadow economy—February 2020”

Figure 16 illustrates this trend, highlighting the surge in the number of accommodations on Airbnb in Italy, which has grown from 52 in 2008 to almost half a million in 2019.

The economic, societal, and family trends outlined, notably pronounced in Italy, somewhat less in Germany, but observed across several other nations in Mediterranean Europe, align with the deterioration of tenants’ positions. These factors underscore the need for housing policies that enhance affordability and stability, instead of rent freezes that undermine the core of property rights, which is protected as a fundamental and, ultimately, a human right in the EU. Based on this contextual analysis and the descriptive analysis of the case law, it becomes apparent that the position of the tenants was worsened by the interplay of macro-economic and legal changes starting in the 1980s.

## Taking stock

In this essay, we have explored a large sample of cases on rent control focusing on three courts: the ECtHR, the Italian Constitutional Court (also in relation to the Italian Cassation Court), and the German FCC. A clear jurisprudential trend emerges: these Courts have protected the right to property against State interference through disproportionate rent control measures. *First,* judges generally assume that the fair or correct housing price is mainly a function of supply and demand laws and market dynamics. Rent controls can introduce minimum thresholds to avoid an increase in rent beyond the market value. *Second,* as a fundamental right, property comprises the right to make profit at its core: at most, it excludes only maximum profit. *Third,* the fair rent price corresponds to the market value or average market values. When legislators depart significantly from the market price, then the burden imposed on landlords is disproportionately high. *Fourth,* landlords and house owners should not carry the burden of a shortage of housing, as there are no positive rights to housing having a horizontal effect on privates.

The contextual examination has revealed that the economic, societal, and familial trajectories of the last 30 years align with the weakening of tenants’ positions. Tenants have unequivocally become more vulnerable contractual counterparts, constituting a phenomenon that, with an eye to the future, poses a challenge to legislators that should endorse more systematic measures addressing the complexity of the housing markets.

## Figures and Tables

**Fig. 17:**
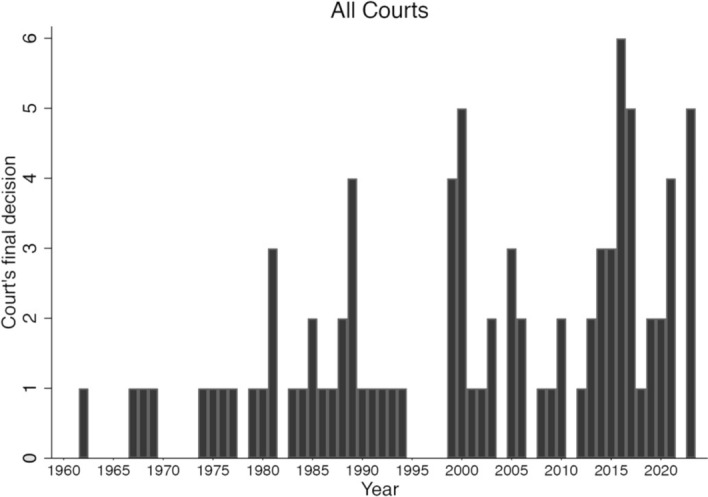
Distribution of cases over time (ECtHR, BVerfGE, and Italian Constitutional Court). Compiled by the authors using own databases

**Fig. 18:**
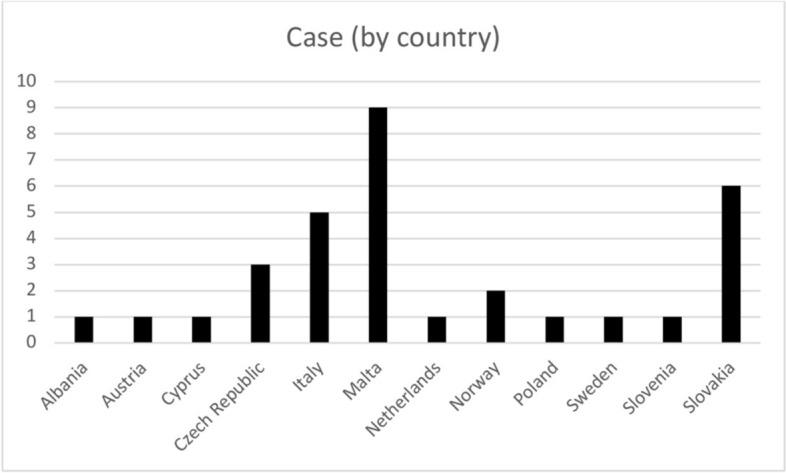
Distribution of cases over time by country (ECtHR). Compiled by the authors using own databases

**Fig. 19:**
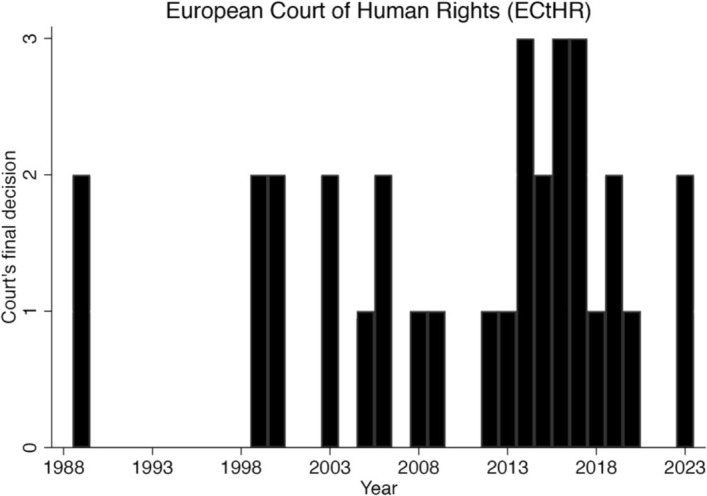
Distribution of cases over time (ECtHR). Compiled by the authors using own databases

**Fig. 20:**
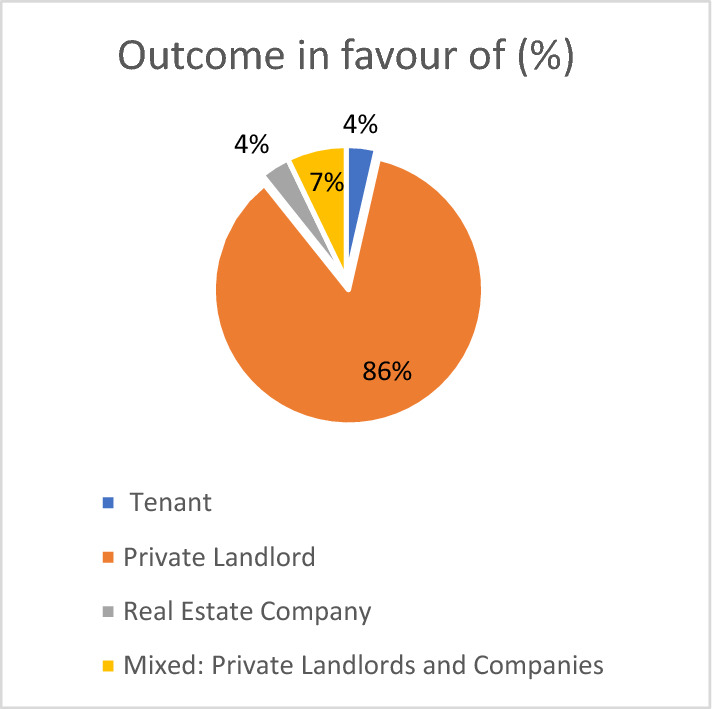
Percentage of cases in favor of the applicants by category (out of the total cases (ECtHR). Compiled by the authors using own databases

**Fig. 21:**
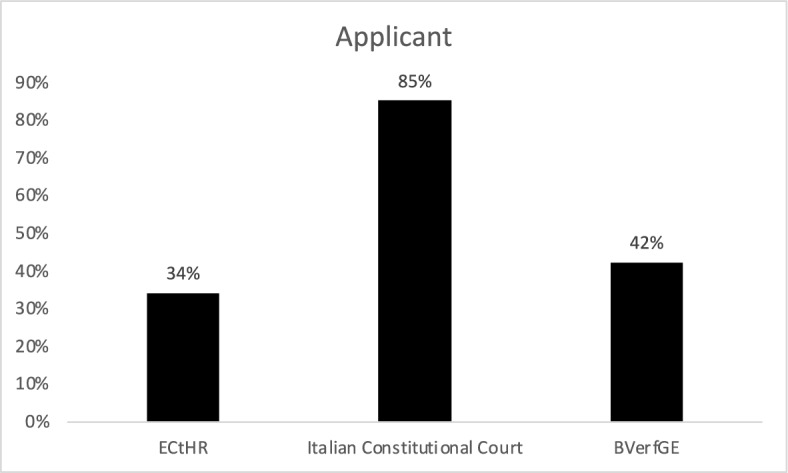
% of cases in which the applicants alleged a violation of the equality clause—all Courts. Compiled by the authors using own databases

**Fig. 22:**
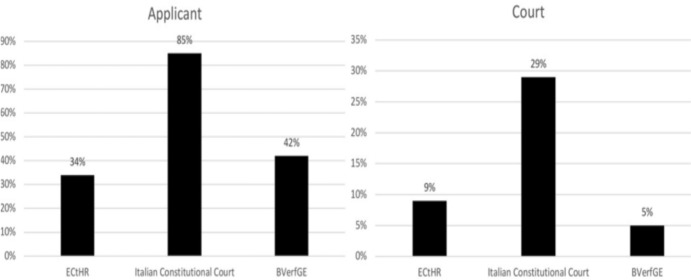
Comparison between the % of cases in which the applicants claimed a violation of the principle of equality and the % of cases in which the Court recognized a violation of the principle of equality. Compiled by the authors using own databases

**Fig. 23:**
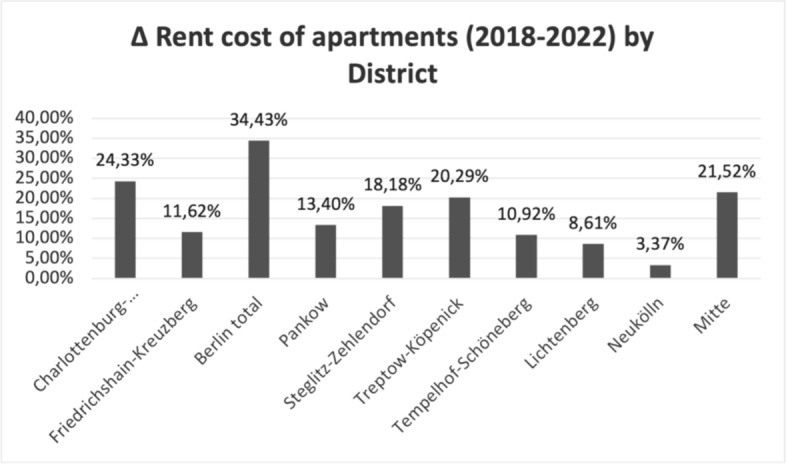
Rent cost of apartments (2018–2022) by district. The source of the data on the median rent of newly rented flats in Berlin (2018–2022) is CBRE Group. The cumulative inflation rate (31/12/2018–31/12/2022) is 15,76% (DECPI2005 index—*Bundesamt*)

## Data Availability

No datasets were generated or analysed during the current study.

## Appendix

### Descriptive statistics on the case law

See Figs. 17, 18, 19, 20, 21 and 22 and Tables

**Table 3:** Winning percentage of applicants per applicant category

Category of applicant	Outcome in favour of		Total	%
	State	Applicant		
Tenant	2	1	3	**33**
Private landlord	1	24	25	**96**
Real estate company	0	1	1	**100**
Mixed: private landlords and companies	1	2	3	**67**

Bold value indicates the percentages

Compiled by the authors using own databases

3,

**Table 4:** Percentage of cases in which the applicants alleged a violation of the equality clause—all Courts

	Yes	Total	Yes (%)
ECtHR	11	32	**34**
Italian Constitutional Court	17	20	**85**
BVerfGE	8	19	**42**

Bold value indicates the percentages

Compiled by the authors using own databases

4 and

**Table 5:** Percentage of cases in which, according to the Court, the principle of equality was violated—all Courts

	Yes	No	Not alleged	Not necessary*	Total	Yes (%)
ECtHR	3	2	18	9	32	**9**
Italian Constitutional Court	5	10	2	0	17	**29**
BVerfGE	1	2	11	5	19	**5**

Bold value indicates the percentages

Compiled by the authors using own databases

* not necessary to examine the question

5.

### List of cases

#### Cases of the ECtHR (32 cases)

1. *Mellacher & Others v. Austria*, Application nos. 10522/83, 11011/84, 11070/84 (December 19, 1989);
2. *Langborger v. Sweden*, Application no. 11179/84 (May 23, 1989);
3. *Larkos v. Cyprus*, Application no. 29515/95 (February 18, 1999);
4. *Immobiliare Saffi v. Italy*, Application no. 22774/93 (July 28, 1999);
5. *A.O v. Italy*, Application no. 22534/93 (October 4, 2000);
6. *G.L. v. Italy*, Application no. 22671/93 (November 3, 2000);
7. *C. SPA v. Italy*, Application no. 34999/97 (July 3, 2003);
8. *Lo Tufo v. Italy*, Application no. 64663/01 (July 21,2005);
9. *Hutten-Czapska v. Poland*, Application no. 35014/97 (June 19, 2006);
10. *Edwards v. Malta*, Application no. 17647/04 (October 24, 2006);
11. *Fleri Soler and Camilleri v. Malta*, Application no. 35349/05 (December 26, 2006)
12. *Ghigo v. Malta*, Application no. 31122/05 (July 17, 2008);
13. *Gnecchi and Barigazzi v. Italy*, Application no. 32006/96 (November 15, 2002);
14. *Amato Gauci v. Malta*, Application no. 47045/06 (December 15, 2009);
15. *Lindheim and Others v. Norway*, Applications nos. 13221/08 and 2139/10 (June 12, 2012);
16. *Nobel and Others v. The Netherlands*, Applications nos. 27126/11, 28084/12, 81046/12 and 81049/12 (July 2, 2013);
17. *Berger-Krall and Others v. Slovenia*, Application no. 14717/04 (October 13, 2014);
18. *Anthony Aquilina v. Malta*, Application no. 3851/12 (December 11, 2014);
19. *R. & L., S.R.O. and Others v. The Czech Republic*, Applications nos. 37926/05, 25784/09, 36002/09, 44410/09 and 65546/09 (July 3, 2014);
20. *Bittó and Others v. Slovakia*, Application No. 30255/09 (January 28, 2014);
21. *Edward Zammit Maempel and Cynthia Zammit Maempel v. Malta*, Application no. 3356/15 (April 15, 2019);
22. *Riedel and Others v. Slovakia*, Applications nos. 44218/07, 54831/07, 33176/08 and 47150/08 (January 10, 2017);
23. *Bukovčanová and Others v. Slovakia*, Application no. 23785/07 (October 5, 2016);
24. *Krahulec v. Slovakia*, Application no. 19294/07 (July 5, 2016);
25. *Rodolfer v. Slovakia*, Application no. 38082/07 (July 5, 2016);
26. *Čapský and Jeschkeová v. the Czech Republic*, Applications nos. 25784/09 and 36002/09 (July 3, 2017);
27. *Mečiar and Others v. Slovakia*, Application no. 62864/09 (April 10, 2017);
28. *Cassar v. Malta*, Application no. 50570/13 (January 30, 2018);
29. *Bradshow and Others v. Malta*, Application no. 37121/15 (January 23, 2019);
30. *Kasmi v. Albania*, Application no. 1175/06 (June 23, 2020);
31. *The Karibu Foundation v. Norway*, Application no. 2317/20 (March 3, 2023);
32. *Pařízek v. the Czech Republic*, Application no. 76286/14 (May 22, 2023).

#### Cases of the Italian Constitutional Court (24 cases)

1. ITA_COST_1976_225
2. ITA_COST_1980_57
3. ITA_COST_1981_111
4. ITA_COST_1983_252
5. ITA_COST_1984_89
6. ITA_COST_1986_108
7. ITA_COST_1987_595
8. ITA_COST_1988_155
9. ITA_COST_1988_217
10. ITA_COST_1989_399
11. ITA_COST_2000_55
12. ITA_COST_2000_482
13. ITA_COST_2003_28
14. ITA_COST_2006_451
15. ITA_COST_2015_169
16. ITA_COST_2016_26
17. ITA_COST_2016_135
18. ITA_COST_2017_87
19. ITA_COST_2020_79
20. ITA_COST_2021_20
21. ITA_COST_2021_128 (eviction moratorium)
22. ITA_COST_2021_213 (eviction moratorium)
23. ITA_COST_2021_205

#### Cases of the Italian Cassation Court (15 cases)

1. ITA_CASS_1992_6245
2. ITA_CASS_1999_182
3. ITA_CASS_2000_15140
4. ITA_CASS_2001_332
5. ITA_CASS_2002_3431
6. ITA_CASS_2005_11603
7. ITA_CASS_2010_9548
8. ITA_CASS_2010_23053
9. ITA_CASS_2013_14867
10. ITA_CASS_2014_15899
11. ITA_CASS_2016_ 25599
12. ITA_CASS_2017_23601
13. ITA_CASS_2023_ 24176
14. ITA_CASS_2023_13879
15. ITA_CASS_2023_24819

#### Cases of the BVerfGE (19 Cases)

1. BVerfGE 14, 263 (Urteil des Ersten Senats vom 07.08.1962—1 BvL 16/60)
2. BVerfGE 21, 73 (Beschluss des Ersten Senats vom 12.01.1967—1 BvR 169/63)
3. BVerfGE 24, 367 (Urteil des Ersten Senats vom 18.12.1968—1 BvR 638/64, 1 BvR 673/64, 1 BvR 200/65, 1 BvR 238/65,1 BvR 249/65)
4. BVerfGE 25, 112 (Beschluss des Ersten Senats vom 15.01.1969—1 BvL 3/66)
5. BVerfGE 37, 132 (Beschluss des Ersten Senats vom 23.04.1974—1 BvR 6/74, 2270/73)
6. BVerfGE 38, 348 (Beschluss des Zweiten Senats vom 04.02.1975—2 BvL 5/74)
7. BVerfGE 46, 325 (Beschluss des Ersten Senats vom 07.12.1977—1 BvR 734/77)
8. BVerfGE 52, 1 (12.06.1979—1 BvL 19/76)
9. BVerfGE 56, 249 (10.03.1981—1 BvR 92/71, 1 BvR 96/71)
10. BVerfGE 58, 300 (Beschluss des Ersten Senats vom 15.07.1981—1 BvL 77/78)
11. BVerfGE 68, 361 (Beschluss des Ersten Senats vom 08.01.1985—1 BvR 792/83, 1 BvR 501/83)
12. BVerfGE 71, 230 (Beschluss des Ersten Senats vom 04.12.1985—1 BvL 23/84, 1 BvL 1/85, 1 BvR 439, 1 BvR 652/84)
13. BVerfGE 79, 292 (Urteil des Ersten Senats vom 14.02.1989—1 BvR 308, 1 BvR 336/88, 1 BvR 356/88)
14. BVerfGE 83, 82 (Beschluss des Ersten Senats vom 13.11.1990—1 BvR 275/90)
15. BVerfGE 89, 1 (Beschluss des Ersten Senats vom 26.05.1993—1 BvR 208/93)
16. BVerfGE 91, 294 (Beschluss des Ersten Senats vom 22.11.1994—1 BvR 351/91)
17. BVerfGE 100, 226 (Beschluss des Ersten Senats vom 02.03.1999—1 BvL 7/91)
18. BVerfGE 143, 246 (Urteil des Ersten Senats vom 06.12.2016—1 BvR 2821/11, 1 BvR 321/12, 1 BvR 1456/12)
19. BVerfGE 157, 223 (Beschluss des Zweitens Senats vom 25.03.2021—2 BvF 1/20, 2 BvL 4/20, 2 BvL 5/20)

### Median rent of newly rented apartments in Berlin, Germany from 2018 to 2022 by district (euro/sq meter) and inflation rate

Except in a few neighborhoods, house prices still rose faster than the rate of inflation. The inflation considered here is the same for all of Germany, while instead there will be some spatial heterogeneities that, at the city level, are negligible. All analyses are done by the Authors on median prices (50% new rental apartments cost higher and 50% lower) (Fig. 23 and Table

**Table 6:** Cumulative Δ rent cost of apartments and cumulative inflation rate by district (2018–2022)

*Charlottenburg-Wilmersdorf*	
Cumulative inflation rate	15.76%
Δ Rent cost of apartments	24.33%
*Friedrichshain-Kreuzberg*	
Cumulative inflation rate	15.76%
Δ Rent cost of apartments	11.62%
*Berlin total*	
Cumulative inflation rate	15.76%
Δ Rent cost of apartments	34.43%
*Pankow*	
Cumulative inflation rate	15.76%
Δ Rent cost of apartments	13.40%
*Steglitz-Zehlendorf*	
Cumulative inflation rate	15.76%
Δ Rent cost of apartments	18.18%
*Treptow-Köpenick*	
Cumulative inflation rate	15.76%
Δ Rent cost of apartments	20.29%
*Tempelhof-Schöneberg*	
Cumulative inflation rate	15.76%
Δ Rent cost of apartments	10.92%
*Lichtenberg*	
Cumulative inflation rate	15.76%
Δ Rent cost of apartments	8.61%
*Neukölln*	
Cumulative inflation rate	15.76%
Δ Rent cost of apartments	3.37%
*Mitte*	
Cumulative inflation rate	15.76%
Δ Rent cost of apartments	21.52%

The source of the data on the median rent of newly rented flats in Berlin (2018–2022) is CBRE Group. The index employed to calculate inflation is the DECPI2005 index (*Bundesamt*). The analyzed period is between 31.12.2018 and 31.12.2022. When the growth rate of house prices (2018–2022) is lower than the inflation rate (2018–2022), it is shown in red

6).

**Table:**

	Charlottenburg-Wilmersdorf	Δ Rent cost of apartments (yearly)**	Inflation rate (yearly)*
2018	12	Base year	Base year
2019	12.65	5.42%	1.54%
2020	12.45	− 1.58%	− 0.28%
2021	13.01	4.50%	5.30%
2022	14.92	14.68%	8.57%

* The index employed to calculate inflation is the DECPI2005 index (Bundesamt). The annual inflation rate is calculated on an annual basis (31/12/year “t-1”—31/12/year “t”)

** The source of the data on the median rent of newly rented flats in Berlin (2018–2022) is CBRE Group

**Table:**

	Friedrichshain-Kreuzberg	Δ Rent cost of apartments (yearly)**	Inflation rate (yearly)*
2018	12.99	Base year	Base year
2019	13	0.08%	1.54%
2020	13.20	1.54%	− 0.28%
2021	13.33	0.98%	5.30%
2022	14.50	8.78%	8.57%

* The index employed to calculate inflation is the DECPI2005 index (Bundesamt). The annual inflation rate is calculated on an annual basis (31/12/year “t-1”—31/12/year “t”)

** The source of the data on the median rent of newly rented flats in Berlin (2018–2022) is CBRE Group

**Table:**

	Berlin total	Δ Rent cost of apartments (yearly)**	Inflation rate (yearly)*
2018	10.34	Base year	Base year
2019	10.44	0.97%	1.54%
2020	10.15	− 2.78%	− 0.28%
2021	10.50	3.45%	5.30%
2022	13.90	32.38%	8.57%

* The index employed to calculate inflation is the DECPI2005 index (Bundesamt). The annual inflation rate is calculated on an annual basis (31/12/year “t-1”—31/12/year “t”)

** The source of the data on the median rent of newly rented flats in Berlin (2018–2022) is CBRE Group

**Table:**

	Pankow	Δ Rent cost of apartments (yearly)**	Inflation rate (yearly)*
2018	10.97	Base year	Base year
2019	10.94	− 0.27%	1.54%
2020	10.50	− 4.02%	− 0.28%
2021	11.59	10.38%	5.30%
2022	12.44	7.33%	8.57%

* The index employed to calculate inflation is the DECPI2005 index (Bundesamt). The annual inflation rate is calculated on an annual basis (31/12/year “t-1”—31/12/year “t”)

** The source of the data on the median rent of newly rented flats in Berlin (2018–2022) is CBRE Group

**Table:**

	Steglitz-Zehlendorf	Δ Rent cost of apartments (yearly)**	Inflation rate (yearly)*
2018	10.34	Base year	Base year
2019	10.67	3.19%	1.54%
2020	10.38	− 2.72%	− 0.28%
2021	11.01	6.07%	5.30%
2022	12.22	10.99%	8.57%

* The index employed to calculate inflation is the DECPI2005 index (Bundesamt). The annual inflation rate is calculated on an annual basis (31/12/year “t-1”—31/12/year “t”)

** The source of the data on the median rent of newly rented flats in Berlin (2018–2022) is CBRE Group

**Table:**

	Treptow-Köpenick	Δ Rent cost of apartments (yearly)**	Inflation rate (yearly)*
2018	9.61	Base year	Base year
2019	9.92	3.23%	1.54%
2020	10	0.81%	− 0.28%
2021	10.29	2.90%	5.30%
2022	11.56	12.34%	8.57%

* The index employed to calculate inflation is the DECPI2005 index (Bundesamt). The annual inflation rate is calculated on an annual basis (31/12/year “t-1”—31/12/year “t”)

** The source of the data on the median rent of newly rented flats in Berlin (2018–2022) is CBRE Group

**Table:**

	Tempelhof-Schöneberg	Δ Rent cost of apartments (yearly)**	Inflation rate (yearly)*
2018	10.26	Base year	Base year
2019	10.52	2.53%	1.54%
2020	10	− 4.94%	− 0.28%
2021	10.29	2.90%	5.30%
2022	11.38	10.59%	8.57%

* The index employed to calculate inflation is the DECPI2005 index (Bundesamt). The annual inflation rate is calculated on an annual basis (31/12/year “t-1”—31/12/year “t”)

** The source of the data on the median rent of newly rented flats in Berlin (2018–2022) is CBRE Group

**Table:**

	Lichtenberg	Δ Rent cost of apartments (yearly)**	Inflation rate (yearly)*
2018	9.64	Base year	Base year
2019	9.26	− 3.94%	1.54%
2020	9.03	− 2.48%	− 0.28%
2021	8.47	− 6.20%	5.30%
2022	10.47	23.61%	8.57%

* The index employed to calculate inflation is the DECPI2005 index (*Bundesamt*). The annual inflation rate is calculated on an annual basis (31/12/year “t-1”—31/12/year “t”)

** The source of the data on the median rent of newly rented flats in Berlin (2018–2022) is CBRE Group

**Table:**

	Neukölln	Δ Rent cost of apartments (yearly)**	Inflation rate (yearly)*
2018	10.09	Base year	Base year
2019	10.06	− 0.30%	1.54%
2020	9.26	− 7.95%	− 0.28%
2021	9.91	7.02%	5.30%
2022	10.43	5.25%	8.57%

* The index employed to calculate inflation is the DECPI2005 index (*Bundesamt*). The annual inflation rate is calculated on an annual basis (31/12/year “t-1”—31/12/year “t”)

** The source of the data on the median rent of newly rented flats in Berlin (2018–2022) is CBRE Group

**Table:**

	Mitte	Δ Rent cost of apartments (yearly)**	Inflation rate (yearly)*
2018	12.50	Base year	Base year
2019	13.42	7.4%	1.54%
2020	13.87	3.4%	− 0.28%
2021	13.91	0.3%	5.30%
2022	15.19	9.2%	8.57%

* The index employed to calculate inflation is the DECPI2005 index *(Bundesamt*). The annual inflation rate is calculated on an annual basis (31/12/year “t-1”—31/12/year “t”)

** The source of the data on the median rent of newly rented flats in Berlin (2018–2022) is CBRE Group

## Footnotes

1 There is a case pending before the U.S. Supreme Court (which has a discretionary docket, so they are still deciding whether to hear the case) that argues that New York City’s Rent Stabilization Law should have a similar market rate-of-return baseline (which it theoretically already does, but the question is how to implement that and the role of courts in substantively overseeing the result of annual rulemaking on rent levels).

2 We have excluded, for example, *Probstmeier v. Germany*, Application no. 20950/92 (July 1, 1997) which deals mainly with procedural issues unrelated to rent controls; *Terra Woningen B.V. v. The Netherlands*, Application no. 20641/92 (December 17, 1996), which deals with more general questions related to housing and urban planning; *Kubát and Others v. the Czech Republic,* Applications nos. 61721/19 and 5 others (June 22, 2023), which deals primarily with proportionate denial of retroactive payment of difference in judges’ salaries.

3 The official website provides a list of all national judges of the Court since 1959, divided by country and election year (https://prd-echr.coe.int/web/echr/composition-of-the-court). Judges come from 47 different countries. We examined every judge’s CV and profile to determine their date of birth and professional background. The list of ECtHR judges comprises legal scholars, professional lawyers and attorneys, politicians (*e.g*. Marc Fischbach, Frederico Bigi), and former national judges. The ECtHR judges not only have different backgrounds, but also come from different legal systems. There are wide age gaps between judges sitting on the same bench (up to 25 years) and the proportion of female judges, although still low, has increased over time.

4 The European Court of Justice (ECJ) is the main judicial body that oversees the correct interpretation of EU law in the Member States over time.

5 The European TENLAW project (ID: 290694) was the first large-scale comparative and European study of tenancy law. The questionnaire, which was completed for all EU countries, also contained questions on the regulation of rent control. The results show that all of the following countries apply second and third generation rent controls: Austria (Hofmann, 2015), Belgium (Haffner & Bounjouh, 2015), Croatia (Jakopič & Žnidarec, 2015), Cyprus (Konistis, 2015), Finland, Sweden (Norberg & Juul-Sandberg, 2016; 2018), France (Hoekstra & Cornette, 2015), Germany (Cornelius & Rzeznik, 2015), Italy (Bianchi, 2015), Hungary (Hegedüs et al., 2015), Latvia (Kolomijceva, 2015), Lithuania (Mikelėnaitė, 2015), Luxembourg (Santos Silva, 2015), the Netherlands (Haffner, van der Veen, & Bounjouh, 2015), Poland (Panek, 2015), Serbia (Petrović, 2015a), Slovakia (Štefanko, 2015), Slovenia (Petrović, 2015b), Spain (Molina Roig, 2015), Switzerland (Wehrmüller, 2015).

6 Rent control regulations are not simply rent ceilings, but complex laws comprising up to a few hundred articles grouped around a rent stabilization mechanism or a rent ceiling, which may itself be fixed or the result of a complex calculation based on synthetic indices. The regulations on rent control create an institutional framework comprising different types of norms: Constitutive rules establishing bodies (*e.g*., oversight boards), rules of conduct for landlords and real estate agencies, sanctions for violating these rules, exemptions and technical rules for determining the controlled price. These norms interact with the relevant provisions regulating rent in the civil law system (*e.g*., rules and principles of the Civil Code regulating property and tenancies) and the fundamental rights enshrined in the Constitution (*e.g*., the fundamental right to property, social rights, etc.), which (metaphorically) form clusters that intersect different levels of the legal system.

7 The measure is literally called “capping limits” (*Kappungsgrenze*). We will return to this point in Sect. 6.2.

8 It is worth stressing that many of the provisions of “Llei 11/2020” have been declared unconstitutional by the Spanish Constitutional Court. See T.C. Sección del Tribunal Constitucional, BOE-A-2022-5807, BOE, No. 84, April 8, 2022, 48,495–48,531.

9 For instance, the French *encadrement de loyer* was introduced by *Loi Alur* in 2014 (Loi n° 2014-366 du 24 mars 2014 *pour l’accès au logement et un urbanisme rénové*) counts no less than 177 articles.

10 Elements 1 to 10 are common to most rent control regulations. They are not separate: a rule establishing a rent control mechanism (*e.g*., a synthetic index) requires another rule establishing an enforcement entity to be *effective* (otherwise there is a technical gap); furthermore, compliance is usually achieved through positive and negative incentives (established by the rent regulations); these incentives, in turn, require a jurisdictional rule establishing the entity (or officials) that will apply the sanction, and so on. We have mapped the *typical* elements of a rent regulation, not all the *necessary and sufficient* conditions that define a rent control in all possible worlds. Some elements may be missing. Nonetheless, we claim that most rent controls are instances of the general type described in this essay.

11 *See* e.g., *Bukovčanová and Others v. Slovakia*, Application no. 23785/07 (October 5, 2016).

12 *See* e.g., *Bittó and Others v. Slovakia*, Application No. 30255/09 (January 28, 2014) §§ 120-125; *Riedel and Others v. Slovakia*, Applications nos. 44218/07, 54831/07, 33176/08 and 47150/08 (April 10, 2017) §§ 35-6; 2; *Larkos v. Cyprus*, Application no. 29515/95 (February 18, 1999) §§ 35ff. The exception is *Bradshow and Others v. Malta*, Application no. 37121/15 (January 23, 2019) §§ 74ff.

13 *A fortiori*, it is impossible to derive a right to a collective negotiation of rent contracts. *See e.g.*, *Langborger v. Sweden*, Application no. 11179/84 (May 23, 1989) § 39.

14 *Hutten-Czapska v. Poland,* Application no. 35014/97 (June 19, 2006); *Rodolfer v. Slovakia,* Application no. 38082/07 (July 5, 2016); *Bukovčanová and Others v. Slovakia*, Application no. 23785/07 (July 5, 2016); *Berger-Krall and Others v. Slovenia*, Application no. 14717/04 (October 13, 2014).

15 *Rodolfer v. Slovakia,* Application no. 38082/07 (July 5, 2016); *Krahulec v. Slovakia*, Application no. 19294/07 (July 5, 2016); *Heldenburg v. The Czech Republic,* Application no. 65546/09 (July 3, 2017); *Case of R. & L., S.R.O. and Others v. The Czech Republic*, Applications nos. 37926/05, 25784/09, 36002/09, 44410/09 and 65546/09 (July 3, 2014); *A.O v. Italy,* Application no. 22534/93 (October 4, 2000), *Lo Tufo v. Italy,* Application no. 64663/01 (July 21, 2005), *G.L. v. Italy*, Application no. 59751/15 (December 10, 2020).

16 *See e.g*., *Cassar v. Malta,* Application no. 50570/13 (January 30, 2018) §§ 84-92; *Edward Zammit Maempel and Cynthia Zammit Maempel v. Malta*, Application no. 3356/15, (April 15, 2019).

17 *See e.g.*, *Kasmi v. Albania,* Application no. 1175/06 (June 23, 2020) § 79.

18 *Krahulec v. Slovakia*, Application no. 19294/07 (July 5, 2016) §§ 32–40; *Lindheim and Others v. Norway*, Applications nos. 13221/08 and 2139/10 (June 12, 2012) §§ 126–135; *Pařízek v. the Czech Republic*, Application no. 76286/14 (May 22, 2023) § 33.

19 *Rodolfer v. Slovakia*, Application no. 38082/07 (July 5, 2016) § 24; *See* also *Bukovčanová and Others v. Slovakia*, Application no. 23785/07 (October 5, 2016) §§ 37–38; *Heldenburg v. The Czech Republic*, Application no. 65546/09 (July 3, 2017) §§ 32–50; *The Karibu Foundation v. Norway*, Application no. 2317/20 (April 3, 2023).

20 *Saliba v. Malta,* Application no. 4251/02 (February 8, 2006) § 37; *Ghigo v. Malta*, Application no. 31122/05 (December 26, 2006) § 60; *Edwards v. Malta*, Application no. 17647/04 (January 24, 2007) § 69. The individual’s fundamental rights at stake are those enshrined in the ECHR, particularly Art. 1 protocol 1, Article 8, and Art. 14 ECHR.

21 *See e.g.*, *Larkos v. Cyprus,* Application no. 29515/95 (February 18, 1999) §§ 33–37.

22 *Zelenchuk and Tsytsyura v. Ukraine*, Applications nos. 846/16 and 1075/16, (August 22, 2018) § 109 (please note: this case concerns agricultural land, not housing); *See* also *Edward Zammit Maempel and Cynthia Zammit Maempel v. Malta*, Application no. 3356/15, (April 15, 2019) § 47.

23 *See e.g*., C. *SPA v. Italy*, Application no. 34999/97 (July 3, 2003) §§ 47–50*; Gnecchi and Barigazzi v. Italy*, Application no. 32006/96 (November 15, 2002) §§ 38–42.

24 It is clear from the case law that the judges do not just want to prevent the maintenance and management costs from being higher than the rent. Rather, the judges assume that the fair price is the market price or the comparative reference price (without considering the maintenance costs, not even for the determination of damages). As a rule, the three courts do not go into detail, by setting different tables, benchmarks, and thresholds for maintenance and management costs. In some cases, both the plaintiffs and the judges refer to the lack of balance between costs and rent to show that the measure is disproportionate. However, the standards for determining a reasonable return by the courts are extremely loose and not evidence based.

25 *Bittó* introduces *inter alia* a particular balancing test for rent controls, that considers: the size of the property in question; the duration of the application of the rent-control scheme in relation to each individual part of the property; the location of the property; the ownership shares of the respective applications in the property, *Bittó and Others v. Slovakia*, Application No. 30255/09 (January 28, 2014) §§ 112–119. This test was applied also in *Riedel and Others v. Slovakia*, Applications nos. 44218/07, 54831/07, 33176/08 and 47150/08 (April 10, 2017) §§ 28–33; *Mečiar and Others v. Slovakia* Application no. 62864/09 (April 10, 2017).

26 As Tarello (2024) notices, the text of the Italian constitution does not contain a definition of “propriety” (*proprietà*), whose content has been defined by the judiciary in stratified way.

27 Pag. 10, par. 11.

28 Law n. 891/1986.

29 *Ex multis*, Corte di Cassazione Decision n. 13879/2023 and n. 13963/2005.

30 Decision n. 24819/2023.

31 Henning (2014) points out that the concept of property, as elaborated by the BVerfGE, encompasses four elements: private use, availability, profit, and stability.

32 This paragraph sets the “content and limits” of the property rights protected by Art. 14 Paragraph 1 GG (Bryde & Wallrabenstein, 2021).

33 This proposition has been reflected in a robust institutional system to protect tenants. The protection takes different forms depending on whether it is an existing or a new tenancy agreement. In the case of existing leases (Section 557 BGB), for example, the German Civil Code (BGB) only allows changes to rents by agreement between both parties and within the limits of formal law. In the event of unilateral rent increases by the landlord, the increases can only become effective with the tenant's consent (Section 558b, Subsection I BGB). If the tenant has not accepted the increase after two months—from the date on which the landlord notified the written declaration—the landlord can only obtain remedy by a decision of a civil court (Section 558b, paragraph 3 BGB). As for new contracts, the rent cap in accordance with Section 556d, Paragraph I of the German Civil Code (BGB) provides important protection for tenants. These strict limitations imposed on landlords show how strongly protected tenants are. This strong protection can also be seen in the case law of the Federal Constitutional Court. The BVerfGE has often interpreted the provisions of tenancy law in favor of tenants. For example, when checking the conditions for a rent increase under the Dismissal Protection Act, the BVerfGE declared that it is constitutional for the legislator to have removed the autonomous determination of the rent by the landlord for existing rents—excluding the so-called “change” notice—because this was compensated for by determining the rent increase according to the local comparable price for comparable apartments (BVerfGE 49, 244: § 15). Finally, the special protection of tenants is also reflected in cases of forced evictions, in which the tenant's rights of possession often prevails on the landlord’s right to regain possession (BVerfGE 83, 82; BVerfGE 89, 1).

34 The duty is clearly entailed by a judicial definition of the private property within the German legal system (BVerfGE 37, 132: 141).

35 According to § 558 BGB, the main criteria for the similarity of living spaces are the following: type, size, equipment, structure, and location.

36 Peter Weber points out that the rent increase according to the local reference rent in existing rental agreements does not represent the market price, but is an “artificial construction” (Weber, 2018). However, the local reference rent is not precisely the actual “market value” rent: rather, it is a prediction on the price development of the rent over time (Weber, 2018).

37 Federal Ministry of Justice (*Bundesministerium der Justiz*), Residential Tenancy Law. Small Guideline (*Wohnraummietrecht. Kleiner Leifaden*).

38 The report is available upon request at www.21re.de. For additional data on Berlin, see also the Berlin Real Estate Market Report H2 2024 available here: www.guthmann.estate/en/market-report/berlin/.

39 Dunning (1977); Helpman (1984); Helpman & Krugman (1985); Brainard (1993).

40 The “cafeteria effect” denotes a set of interactions among creative persons with diverse backgrounds that yields, on the one hand, innovation through tacit information exchange, and, on the other, higher competition on the job market.

41 This is shown by a survey conducted by Statista, which revealed that 71% of respondents consider the Berlin rent cap to be a positive government measure (Statista, 2020) A large proportion of public opinion also views rent control measures such as the one in Berlin positively, as shown by another survey conducted by Statista, which revealed that 52.7% (32.8% said “yes, absolutely” and 19.9% “rather yes”) of respondents were in favor of a nationwide expansion of the rent control, while 35.2% (22% “no, definitely not” and 13.2% “rather no”) were against it (Statista, 2019). To be sure, the survey should be taken with a pinch of salt due to well-known issues concerning sampling strategies.

42 As a rule, the BVerfGE interprets legal provisions that regulate competences according to their wording and their historical meaning. When interpreting competence-rules, the BVerfGE takes seriously their historical origin and the so-called “State practice”. Here, the “State practice” refers to the previous applications of a particular competence rule by the legislatures (namely, a rule following behavior). The BVerfGE derives reads the competence-rules in light of the past practices underpinning the application of the rule by the State organs (BVerfGE 61, 149; Nispel, 2023).

43 BVerfGE 157, 223: 183.

44 BVerfGE 157, 223: 183.

45 This can be seen in the results published by the City of Berlin in: *Der Landeswahleiter für Berlin* (2021). *Volksentscheid “Deutsche Wohnen & Co enteignen”*.

46 The effects and process of this “nationalization” are described in the document published by the Expert Commission which was requested by the Senate of Berlin: *Expertenkommission zum Volkentscheid*, *Vergesellschaftung großer Wohnungsunternehmen*, Abschlussbericht Juni 2023.

47 The acronym Istat denotes the Italian National Institute of Statistics.

48 The acronym INAPP stands for the National Institute for the Analysis of Public Policies (Fadda, 2022).

49 An atypical employment contract encompasses any form of employment agreement that deviates from the standard, full-time and indefinite subordinate employment contract.

50 The acronym OECD stands for the Organization for Economic Co-operation and Development. The dataset used by the OECD refers to wages measured in USD constant prices using the 2016 base year and Purchasing Power Parities (PPP).

51 The Gini index is widely used by economists to measure inequality and can take values ranging from 0 (extreme equality in distribution) to 1 (extreme inequality).

52 Inflation is a “regressive tax”, generally impacting the less affluent disproportionately compared to the more affluent.

53 Eurostat is the statistical office of the European Union (EU).

54 See, among others, Jewkes and Delgadillo (2010) and Salvi del Pero et al. (2016).

55 The report is available upon request at www.idealista.it.
